# Supplementary material for: Histone modifications during the life cycle of the brown alga Ectocarpus
Source: Genome Biol. 2021 Jan 4;22:12. doi: 10.1186/s13059-020-02216-8 (PMC7784034; doi:10.1186/s13059-020-02216-8)
Supplement: Supplementary file 2 — Additional file 2: Figure S1. Histone gene clusters in the Ectocarpus genome. [file 13059_2020_2216_MOESM2_ESM.pdf]

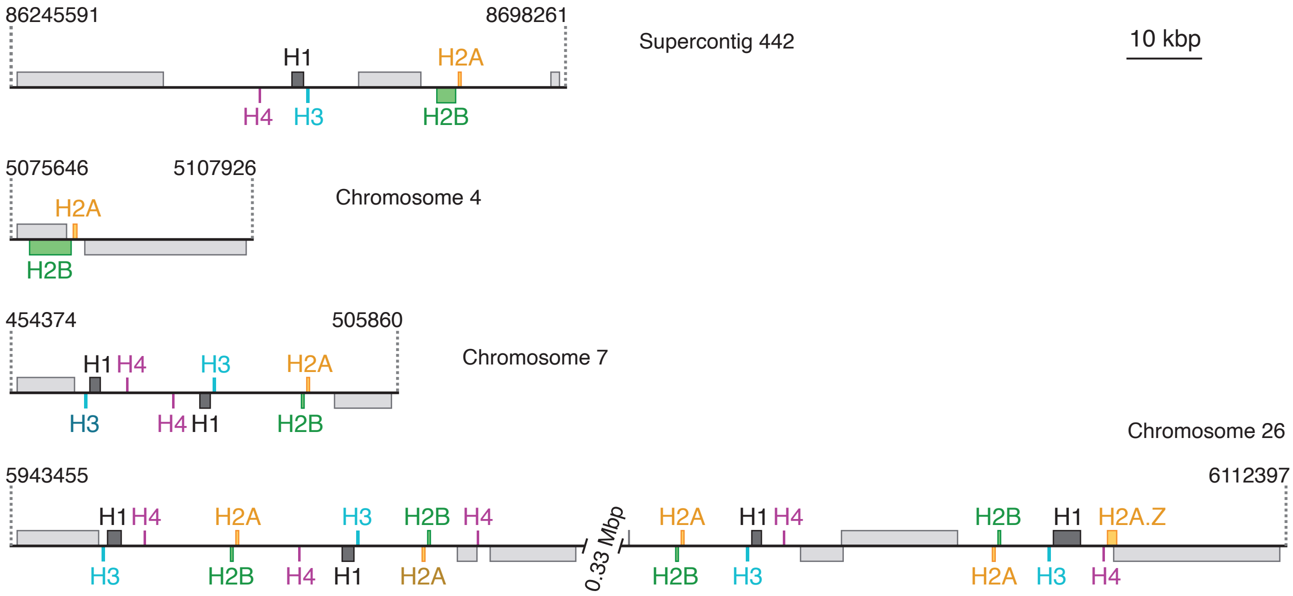

Fig. S1 Histone gene clusters in the *Ectocarpus* genome. Only regions with two or more histone genes are shown. Coding regions from the start to the stop codon are shown as boxes. Histone genes are colour coded, flanking, non-histone genes are in grey. Genes above the line are transcribed to the right, genes below the line to the left. Dotted lines indicate chromosomal or scaffold coordinates.
