# Supplementary material for: Histone modifications during the life cycle of the brown alga Ectocarpus
Source: Genome Biol. 2021 Jan 4;22:12. doi: 10.1186/s13059-020-02216-8 (PMC7784034; doi:10.1186/s13059-020-02216-8)
Supplement: Supplementary file 3 — Additional file 3: Figure S2. Representative MS/MS spectra showing the identification of histone modifications. [file 13059_2020_2216_MOESM3_ESM.pptx]

## Slide 1
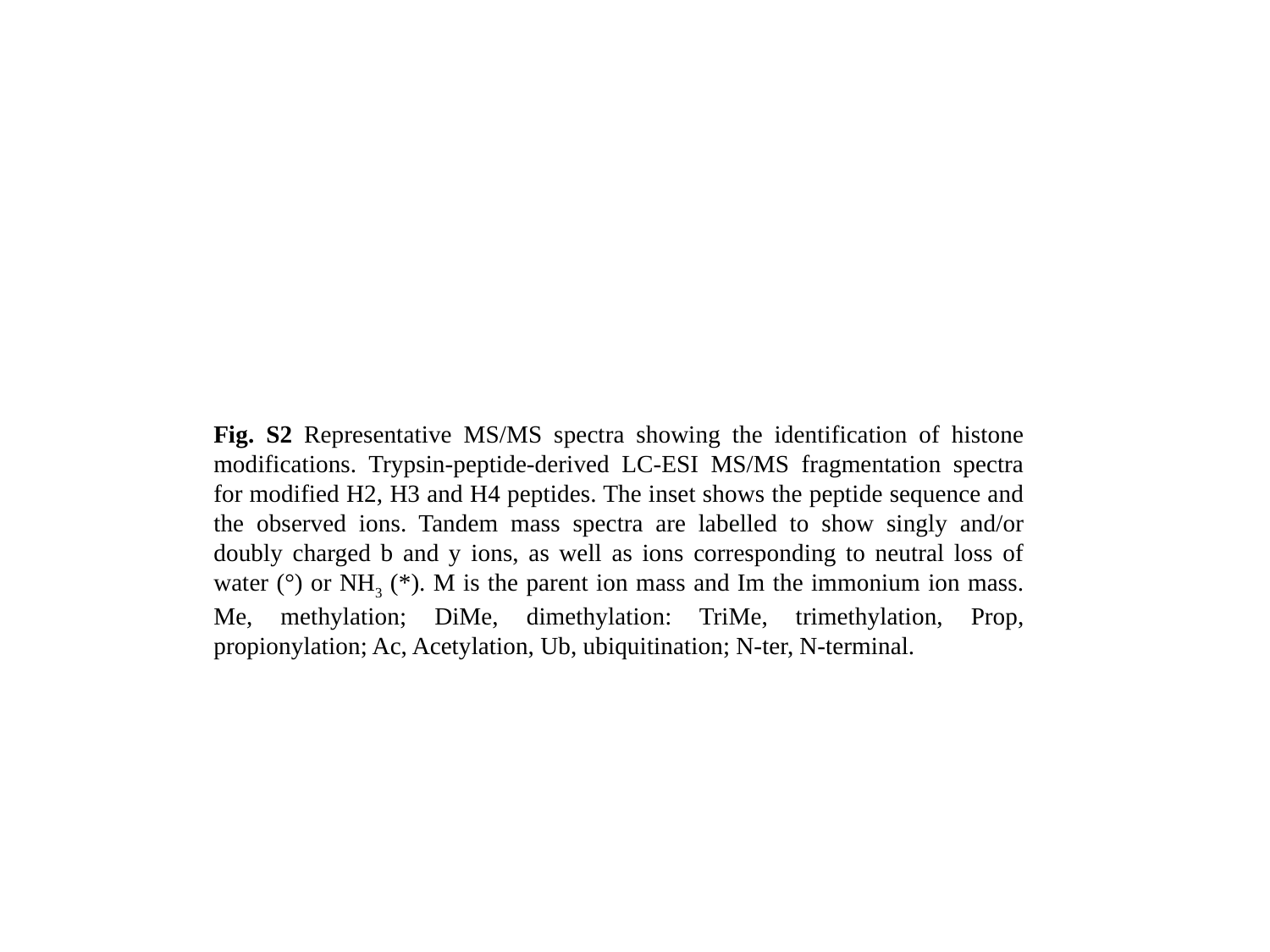

Fig. S2 Representative MS/MS spectra showing the identification of histone modifications. Trypsin-peptide-derived LC-ESI MS/MS fragmentation spectra for modified H2, H3 and H4 peptides. The inset shows the peptide sequence and the observed ions. Tandem mass spectra are labelled to show singly and/or doubly charged b and y ions, as well as ions corresponding to neutral loss of water (°) or NH3 (*). M is the parent ion mass and Im the immonium ion mass. Me, methylation; DiMe, dimethylation: TriMe, trimethylation, Prop, propionylation; Ac, Acetylation, Ub, ubiquitination; N-ter, N-terminal.

## Slide 2
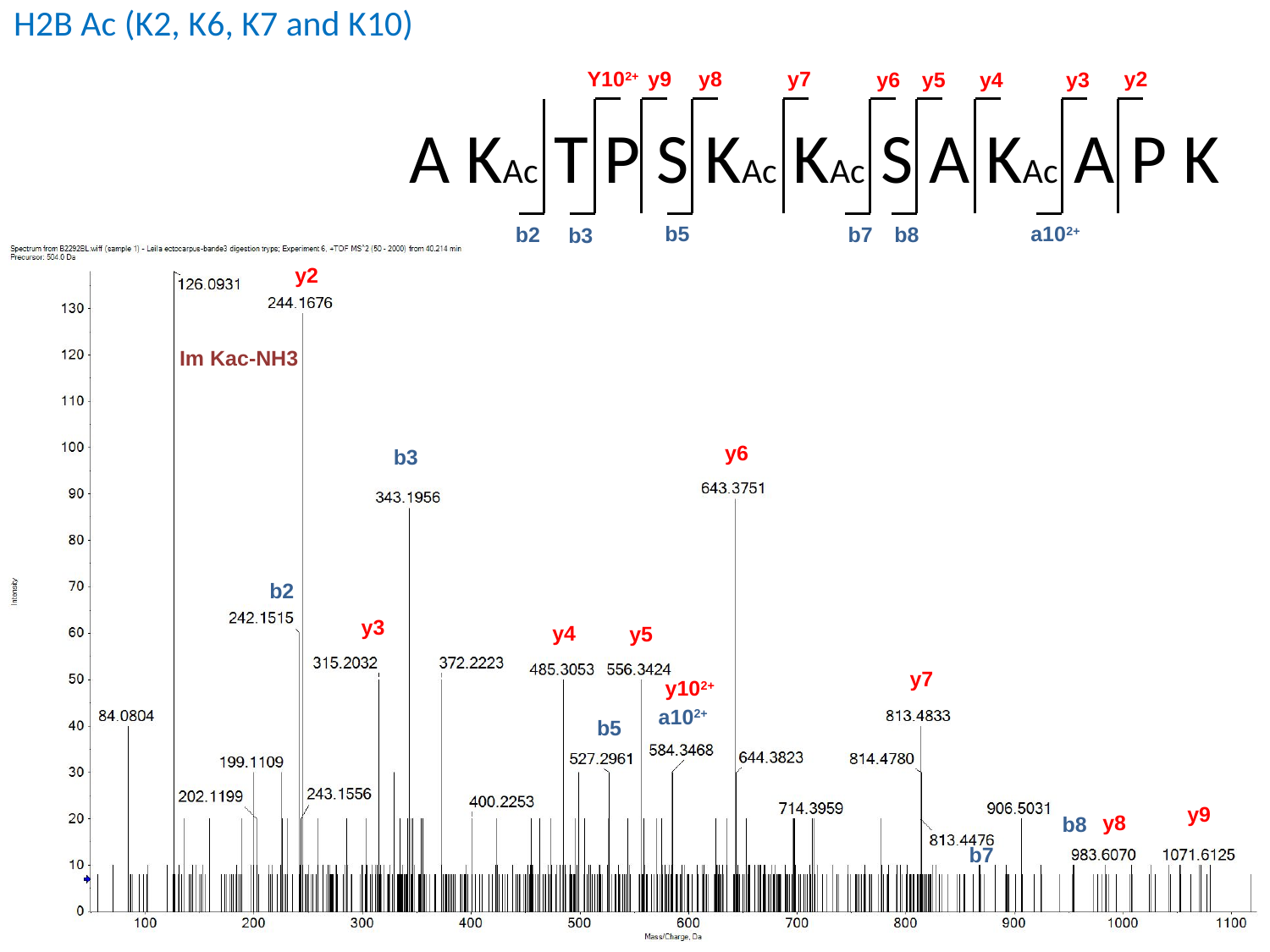

H2B Ac (K2, K6, K7 and K10)
y7
Y102+
y8
y2
y9
y6
y5
y4
y3
A KAc T P S KAc KAc S A KAc A P K
a102+
b5
b7
b2
b8
b3
y2
Im Kac-NH3
y6
b3
b2
y3
y4
y5
y7
a102+
b5
y9
y8
b8
b7
y102+

## Slide 3
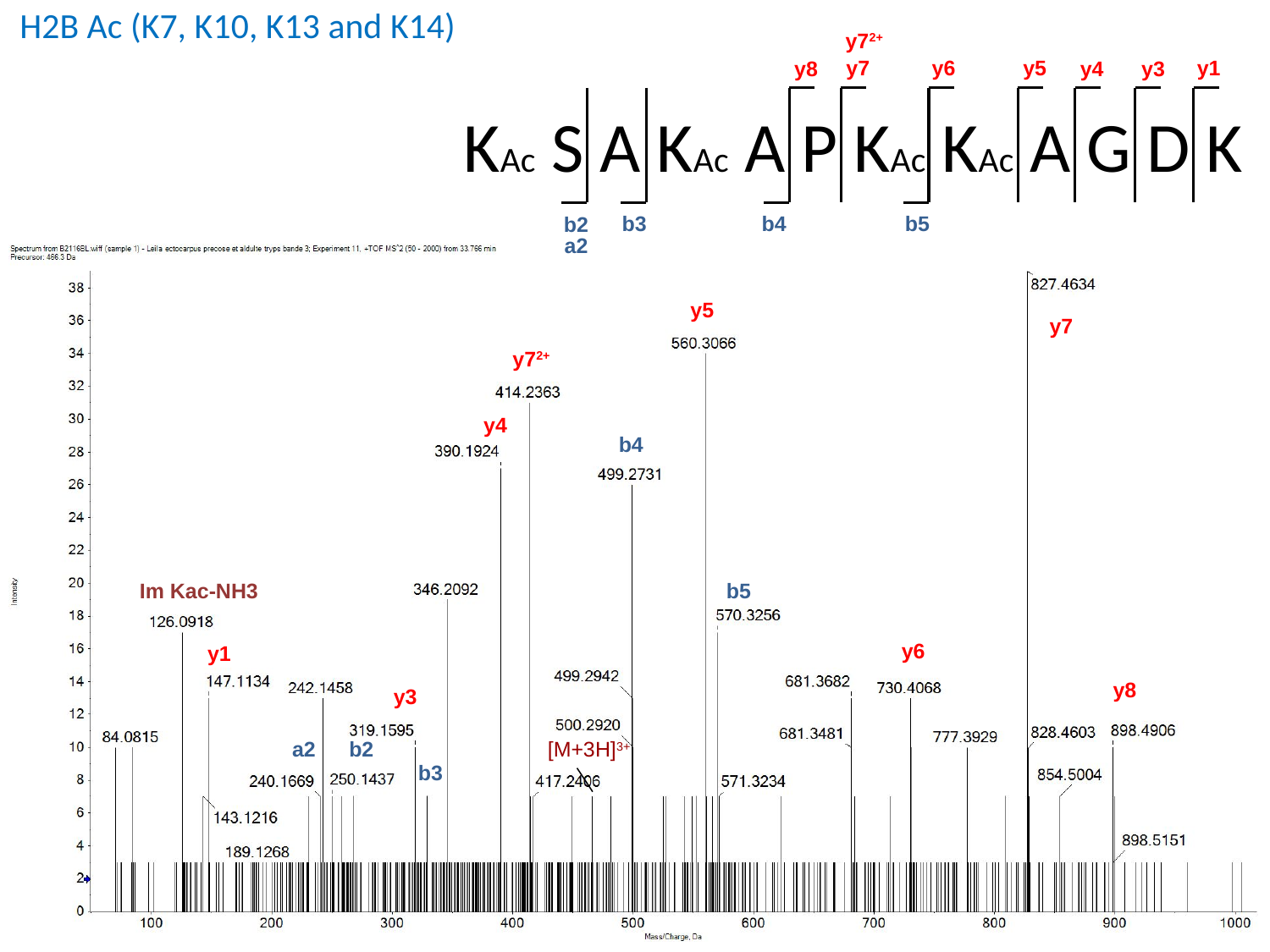

H2B Ac (K7, K10, K13 and K14)
y72+
y7
y1
y5
y6
y8
y4
y3
KAc S A KAc A P KAc KAc A G D K
b4
b3
b5
b2
a2
y5
y7
y72+
y4
b4
b5
Im Kac-NH3
y6
y1
y8
y3
[M+3H]3+
a2
b2
b3

## Slide 4
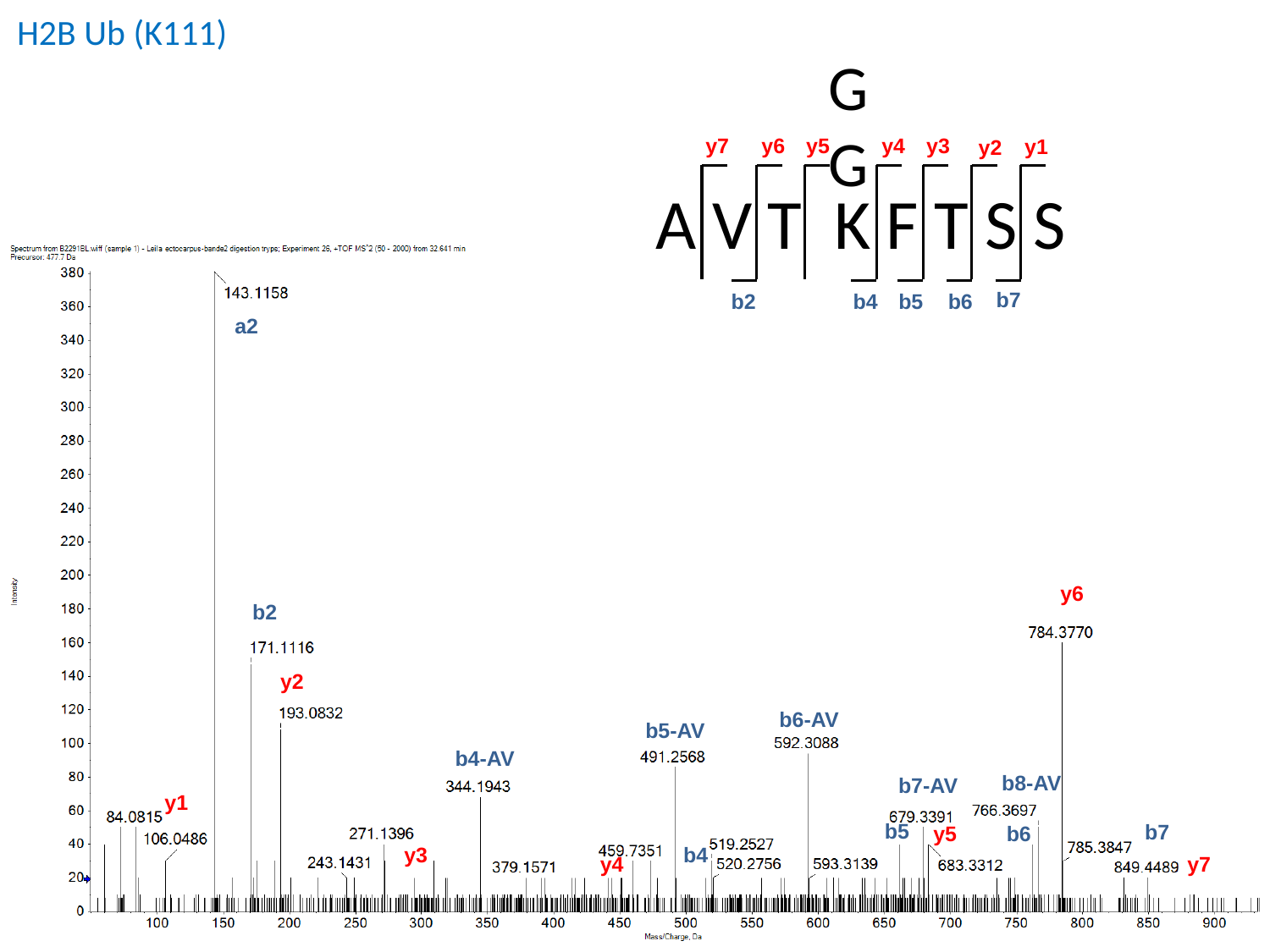

H2B Ub (K111)
GG
y7
y5
y4
y6
y3
y1
y2
A V T K F T S S
b7
b2
b4
b5
b6
a2
y6
b2
y2
b6-AV
b5-AV
b4-AV
b8-AV
b7-AV
y1
b5
b7
b6
y5
y3
b4
y4
y7

## Slide 5
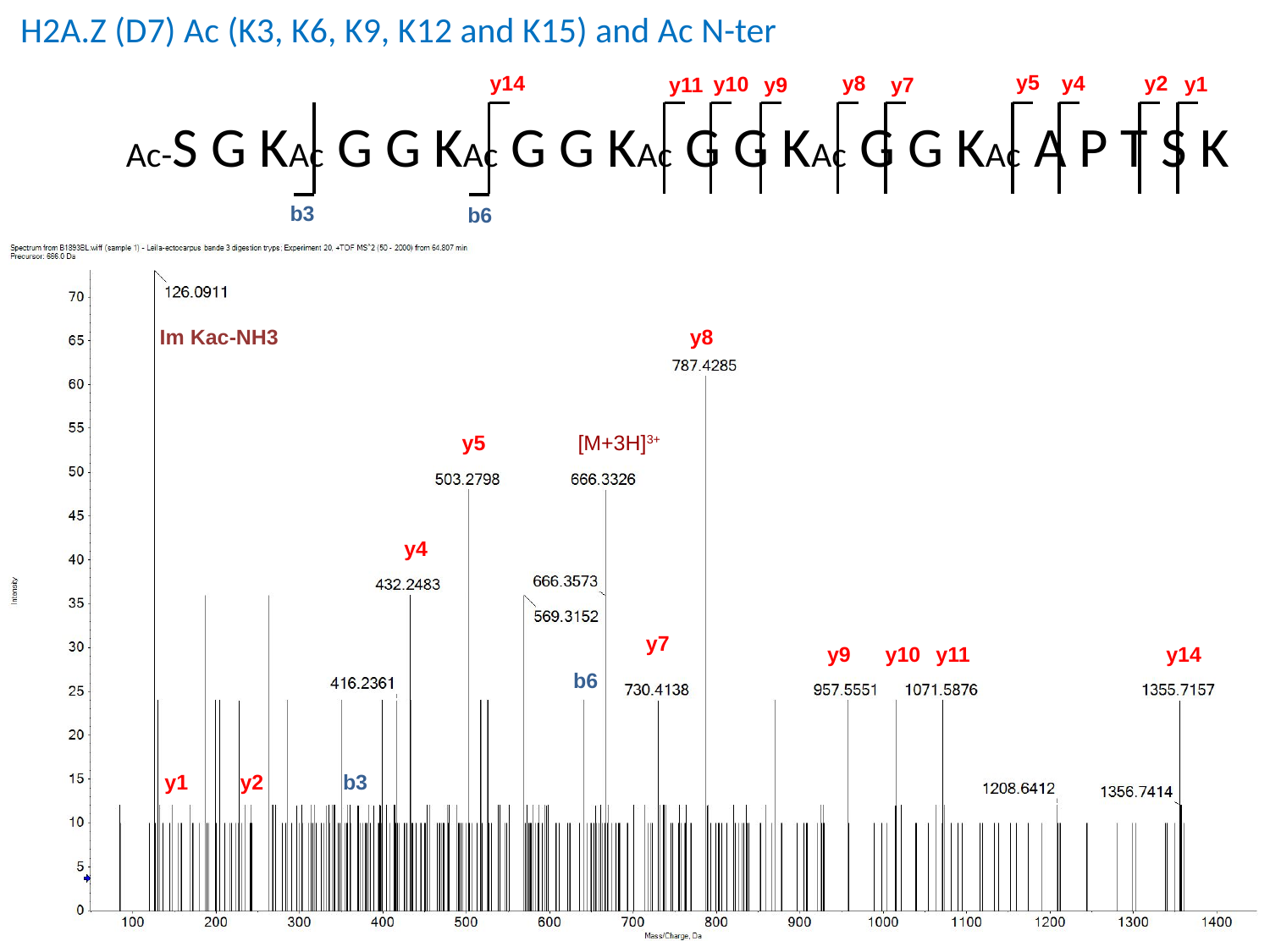

H2A.Z (D7) Ac (K3, K6, K9, K12 and K15) and Ac N-ter
y5
y14
y4
y8
y2
y1
y10
y9
y7
y11
b3
b6
Ac-S G KAc G G KAc G G KAc G G KAc G G KAc A P T S K
Im Kac-NH3
y8
[M+3H]3+
y5
y4
y7
y14
y11
y10
y9
b6
b3
y2
y1

## Slide 6
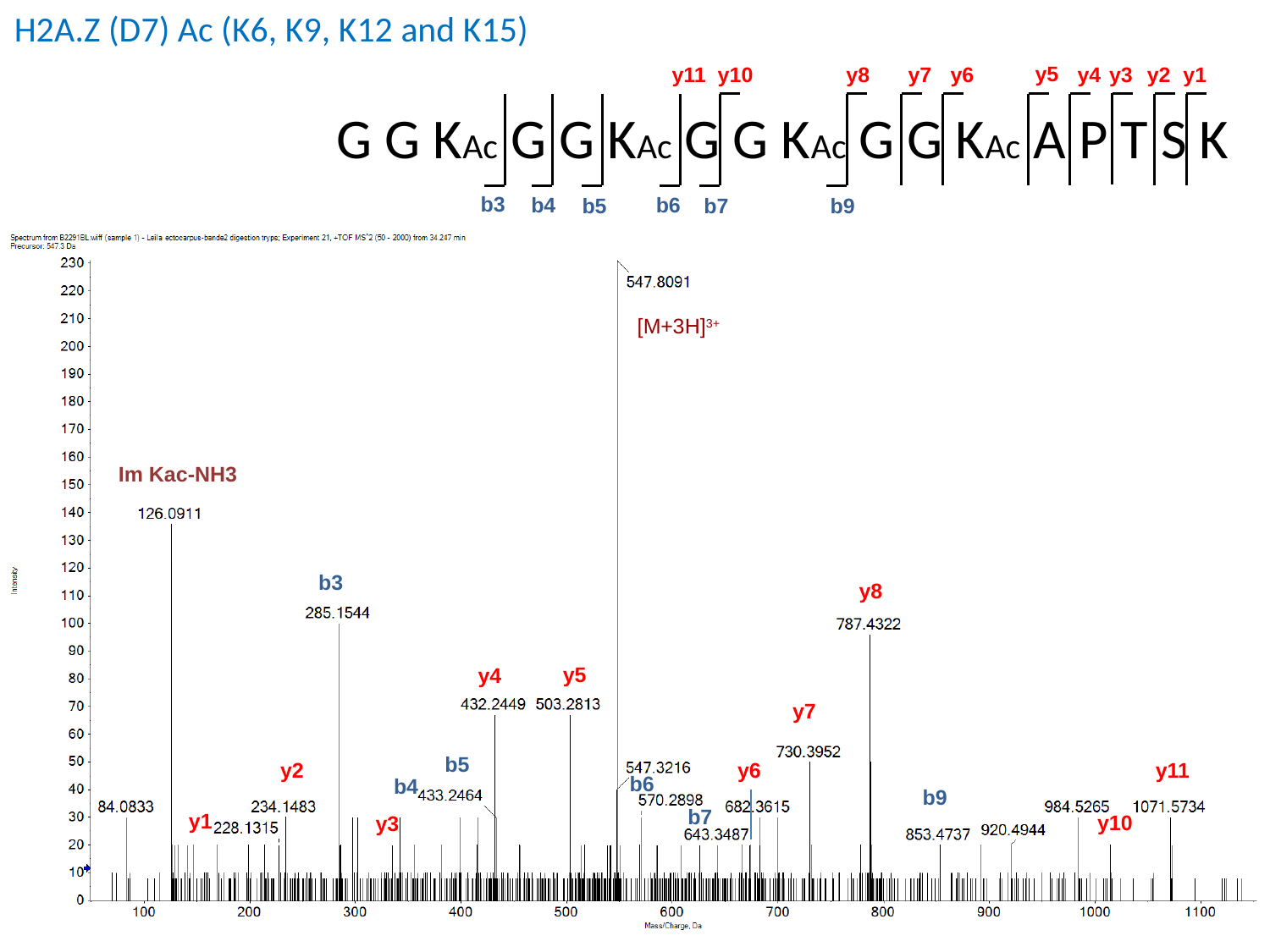

H2A.Z (D7) Ac (K6, K9, K12 and K15)
y5
y10
y4
y3
y6
y1
y11
y8
y7
y2
G G KAc G G KAc G G KAc G G KAc A P T S K
b3
b4
b6
b7
b5
b9
[M+3H]3+
Im Kac-NH3
b3
y8
y5
y4
y7
b5
y2
y6
y11
b6
b4
b9
b7
y1
y10
y3

## Slide 7
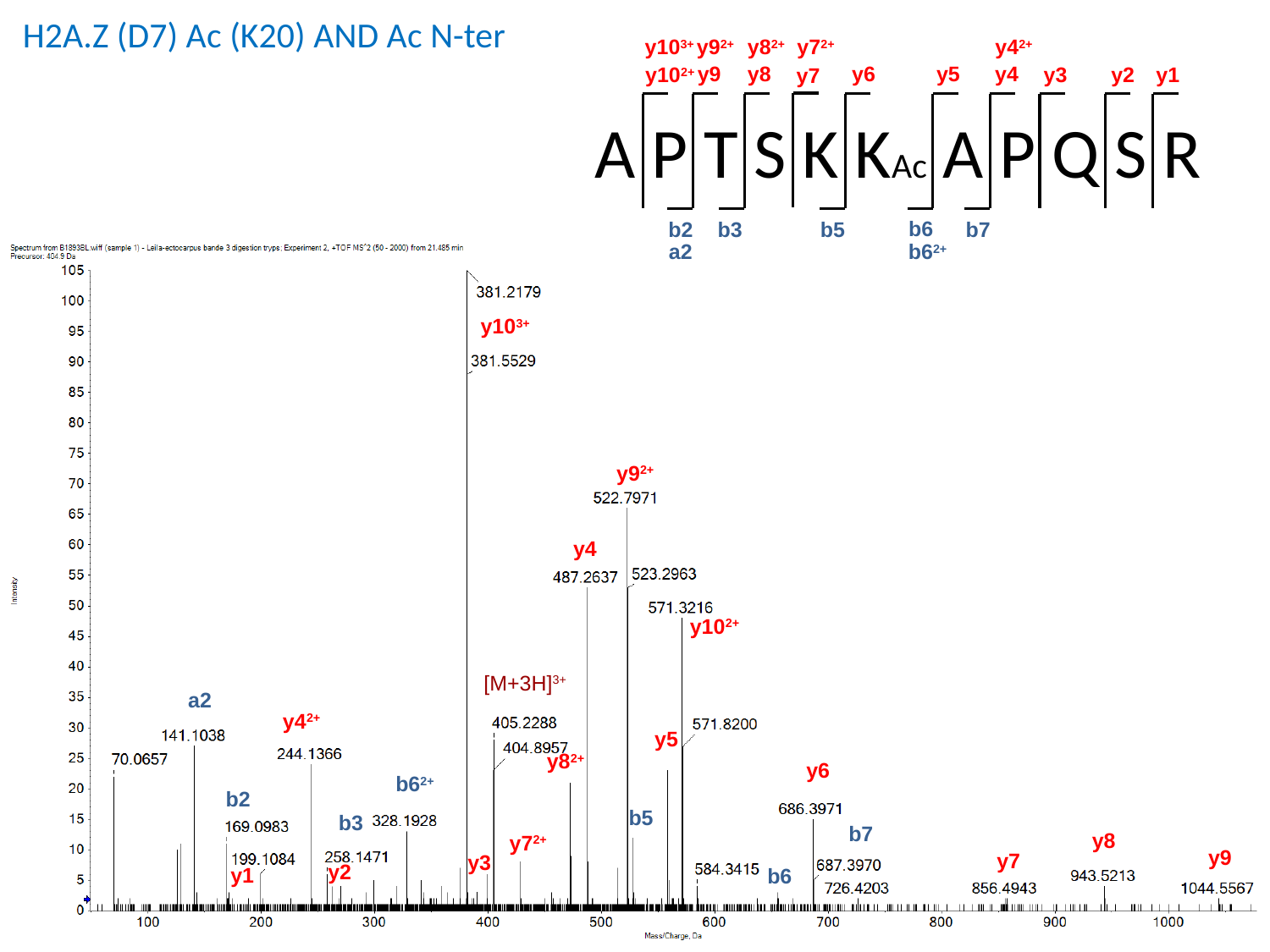

H2A.Z (D7) Ac (K20) AND Ac N-ter
y103+
y92+
y82+
y72+
y42+
y8
y6
y9
y5
y4
y102+
y3
y2
y1
y7
A P T S K KAc A P Q S R
b6
b3
b2
b7
b5
a2
b62+
y103+
y92+
y4
y102+
[M+3H]3+
a2
y42+
y5
y82+
y6
b62+
b2
b5
b3
b7
y8
y72+
y9
y7
y3
y2
y1
b6

## Slide 8
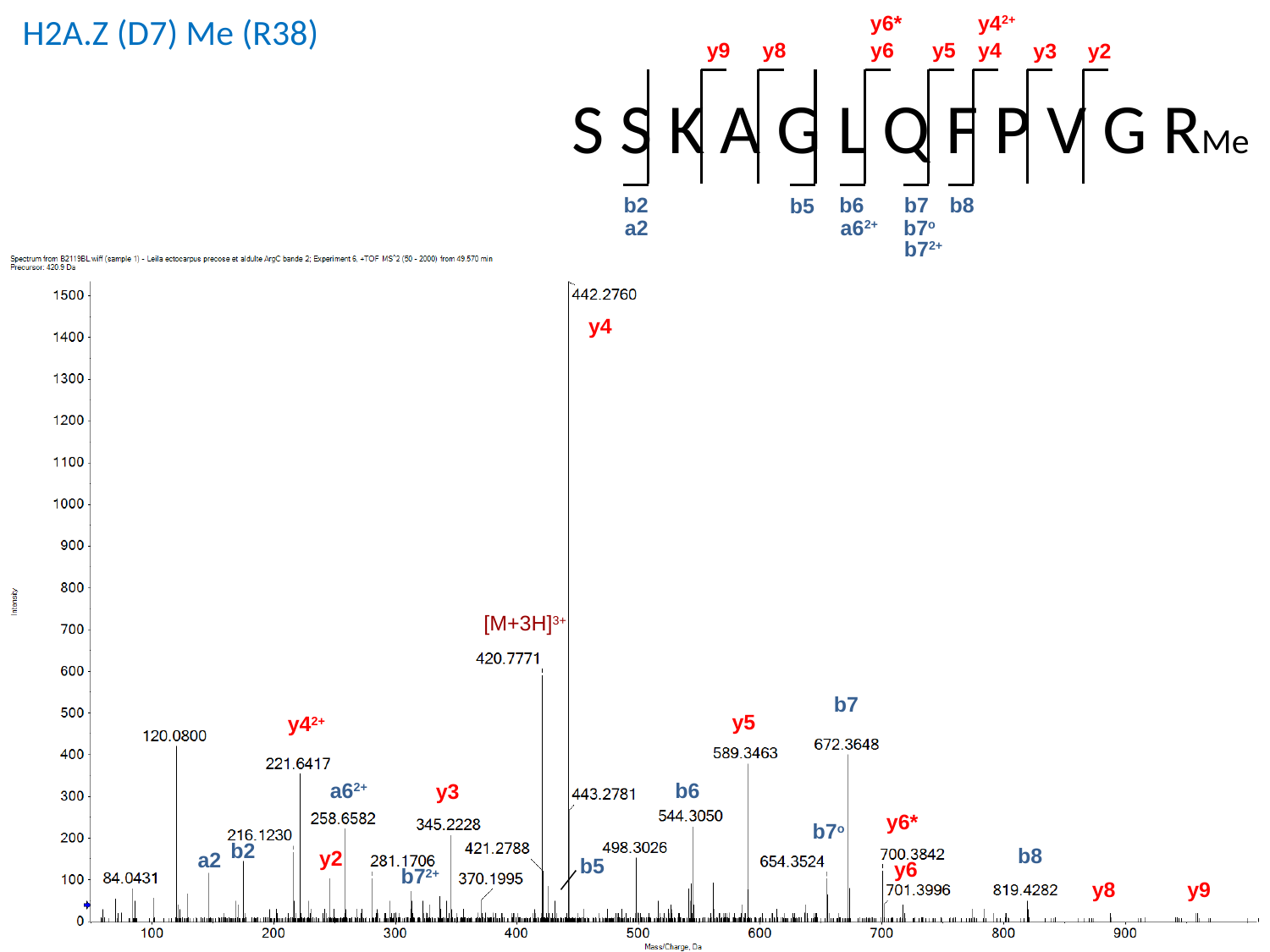

y6*
y42+
y9
y8
y6
y5
y4
y3
y2
S S K A G L Q F P V G RMe
b8
b7
b6
b2
b5
a2
a62+
b7o
b72+
H2A.Z (D7) Me (R38)
y4
[M+3H]3+
b7
y5
y42+
a62+
b6
y3
y6*
b7o
b2
b8
y2
a2
b5
y6
b72+
y9
y8

## Slide 9
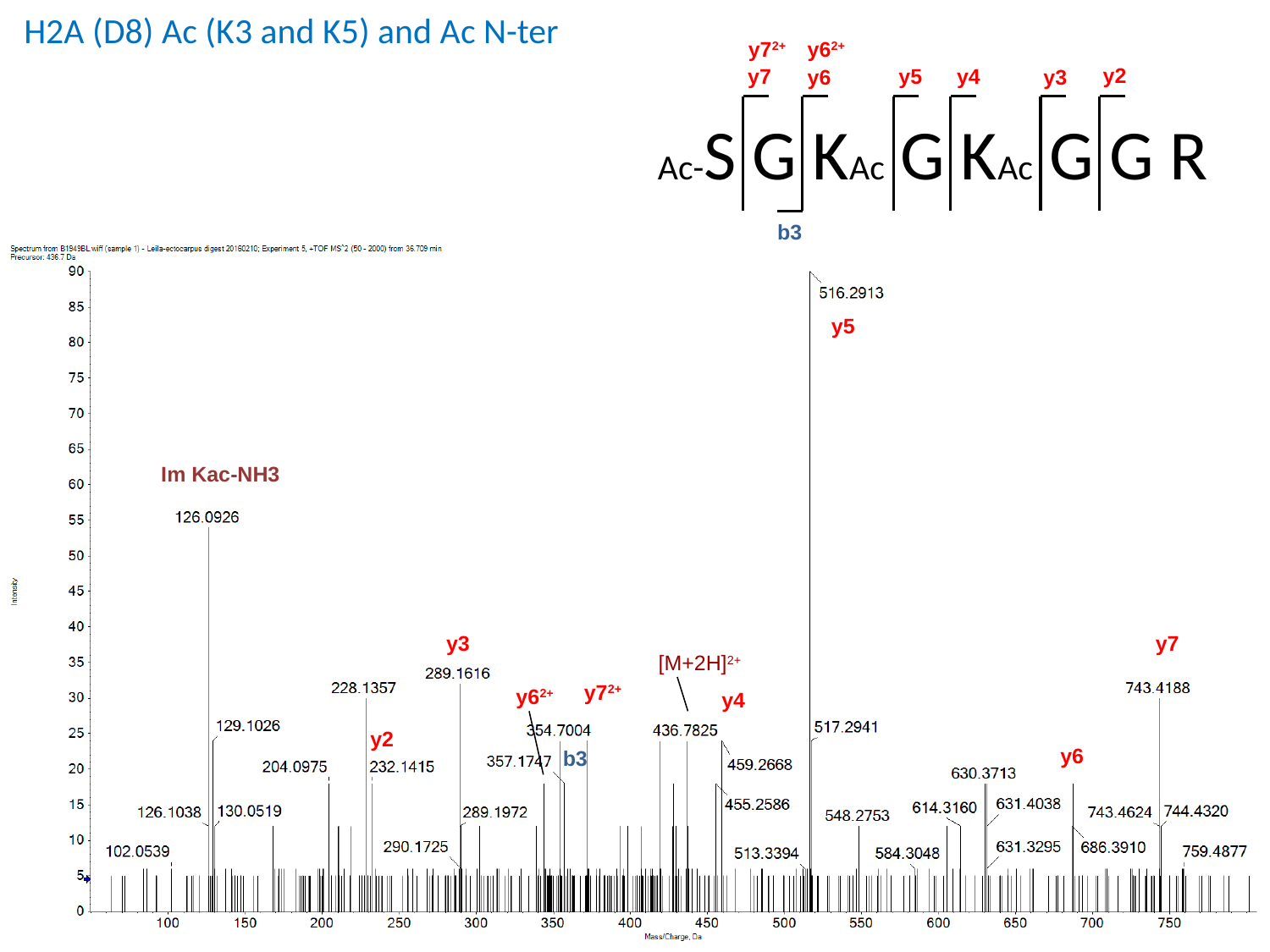

H2A (D8) Ac (K3 and K5) and Ac N-ter
y72+
y62+
y2
y7
y5
y4
y6
y3
Ac-S G KAc G KAc G G R
b3
y5
Im Kac-NH3
y3
y7
[M+2H]2+
y72+
y62+
y4
y2
y6
b3

## Slide 10
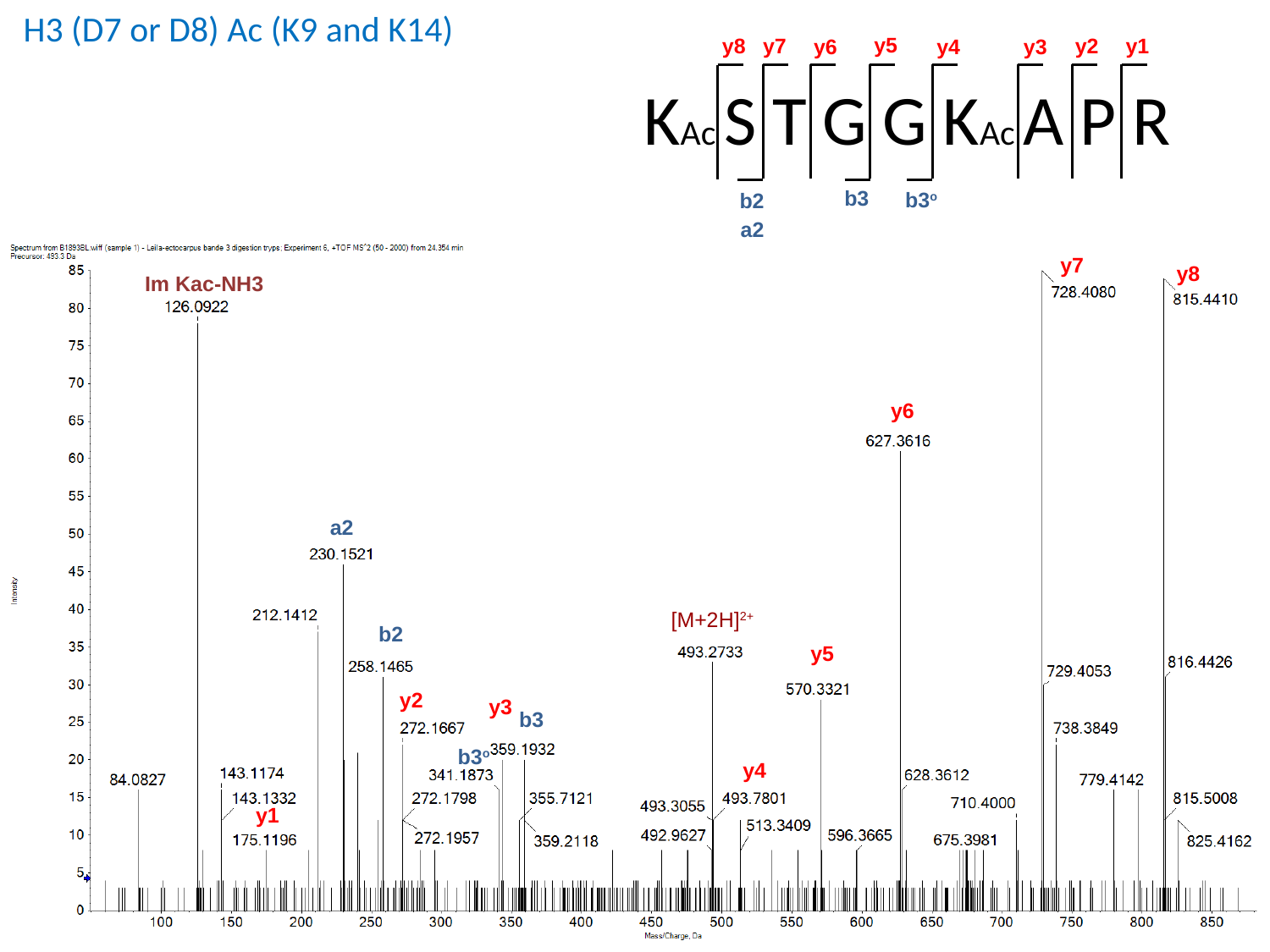

H3 (D7 or D8) Ac (K9 and K14)
y5
y7
y2
y1
y8
y4
y6
y3
KAc S T G G KAc A P R
b3
b3o
b2
a2
y7
y8
Im Kac-NH3
y6
a2
[M+2H]2+
b2
y5
y2
y3
b3
b3o
y4
y1

## Slide 11
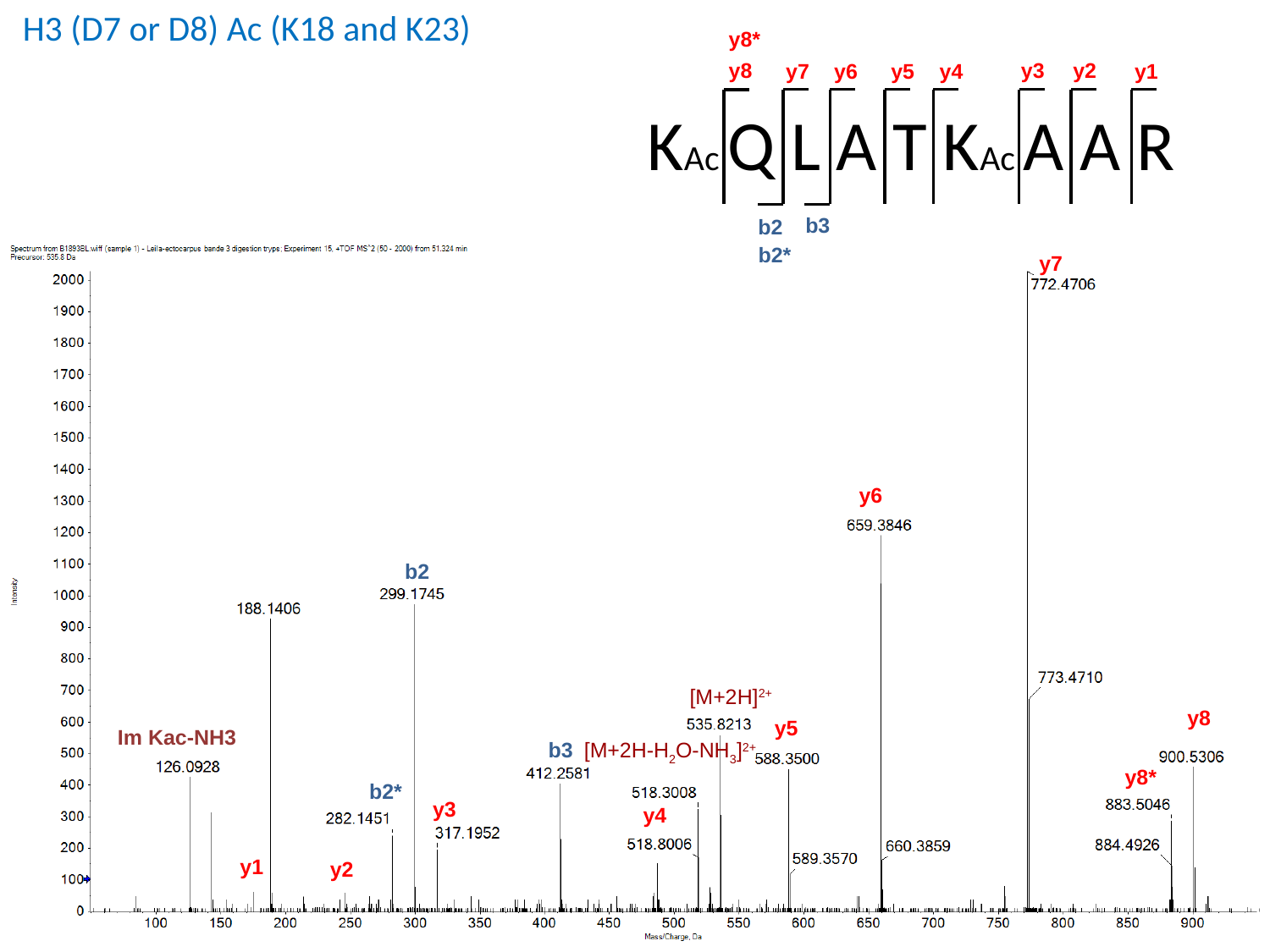

H3 (D7 or D8) Ac (K18 and K23)
y8*
y8
y3
y2
y4
y7
y5
y6
y1
KAc Q L A T KAc A A R
b3
b2
b2*
y7
y6
b2
[M+2H]2+
y8
y5
Im Kac-NH3
b3
[M+2H-H2O-NH3]2+
y8*
b2*
y3
y4
y1
y2

## Slide 12
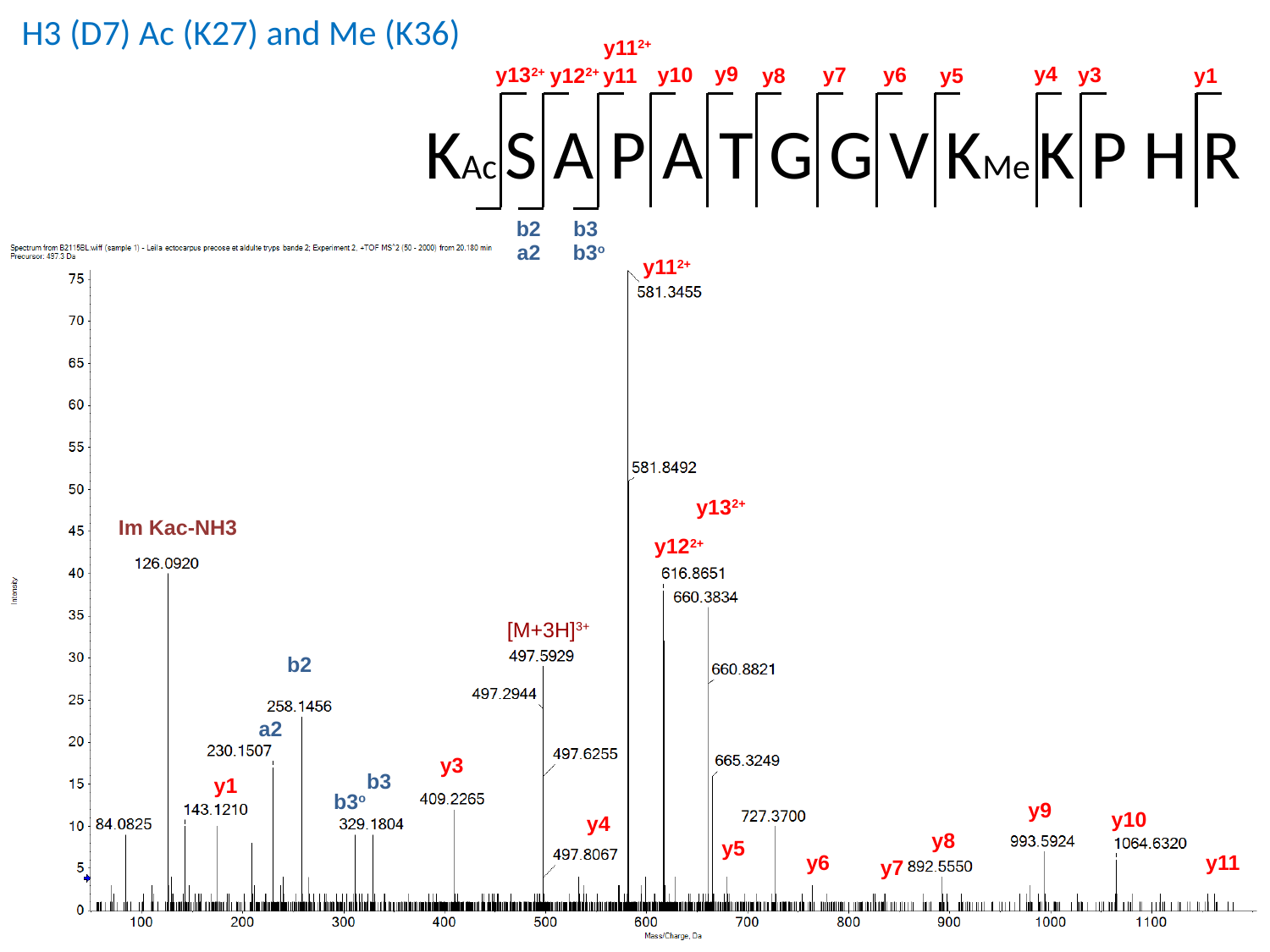

H3 (D7) Ac (K27) and Me (K36)
y112+
y9
y4
y3
y6
y10
y132+
y7
y122+
y11
y5
y8
y1
KAc S A P A T G G V KMe K P H R
b3
b2
a2
b3o
y112+
y132+
Im Kac-NH3
y122+
[M+3H]3+
b2
a2
y3
b3
y1
b3o
y9
y10
y4
y8
y5
y11
y6
y7

## Slide 13
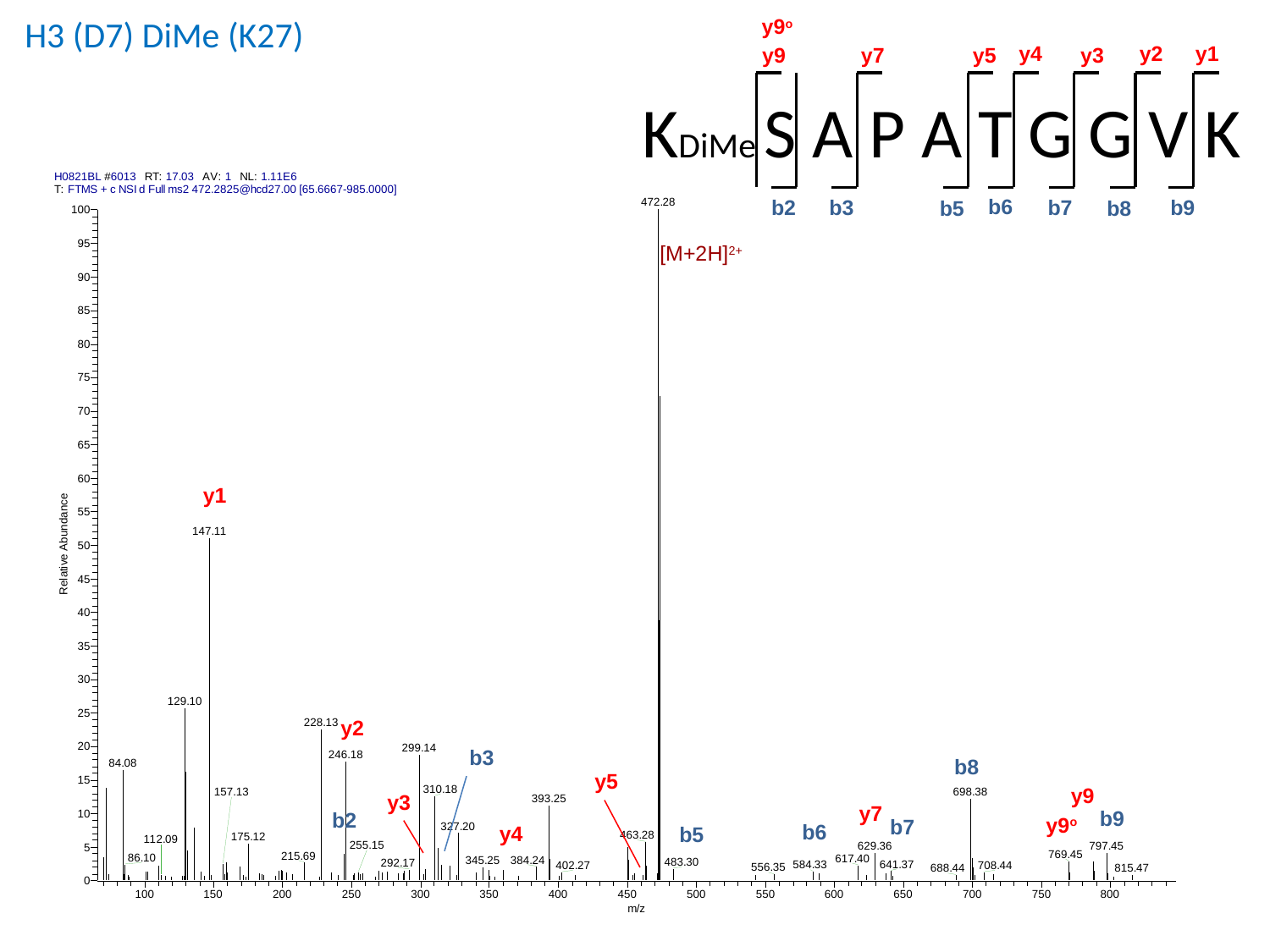

H3 (D7) DiMe (K27)
y9o
y4
y2
y1
y9
y5
y3
y7
KDiMe S A P A T G G V K
b6
b7
b2
b9
b3
b5
b8
[M+2H]2+
y1
y2
b3
b8
y5
y9
y3
y7
b9
b2
y9o
b7
b6
y4
b5

## Slide 14
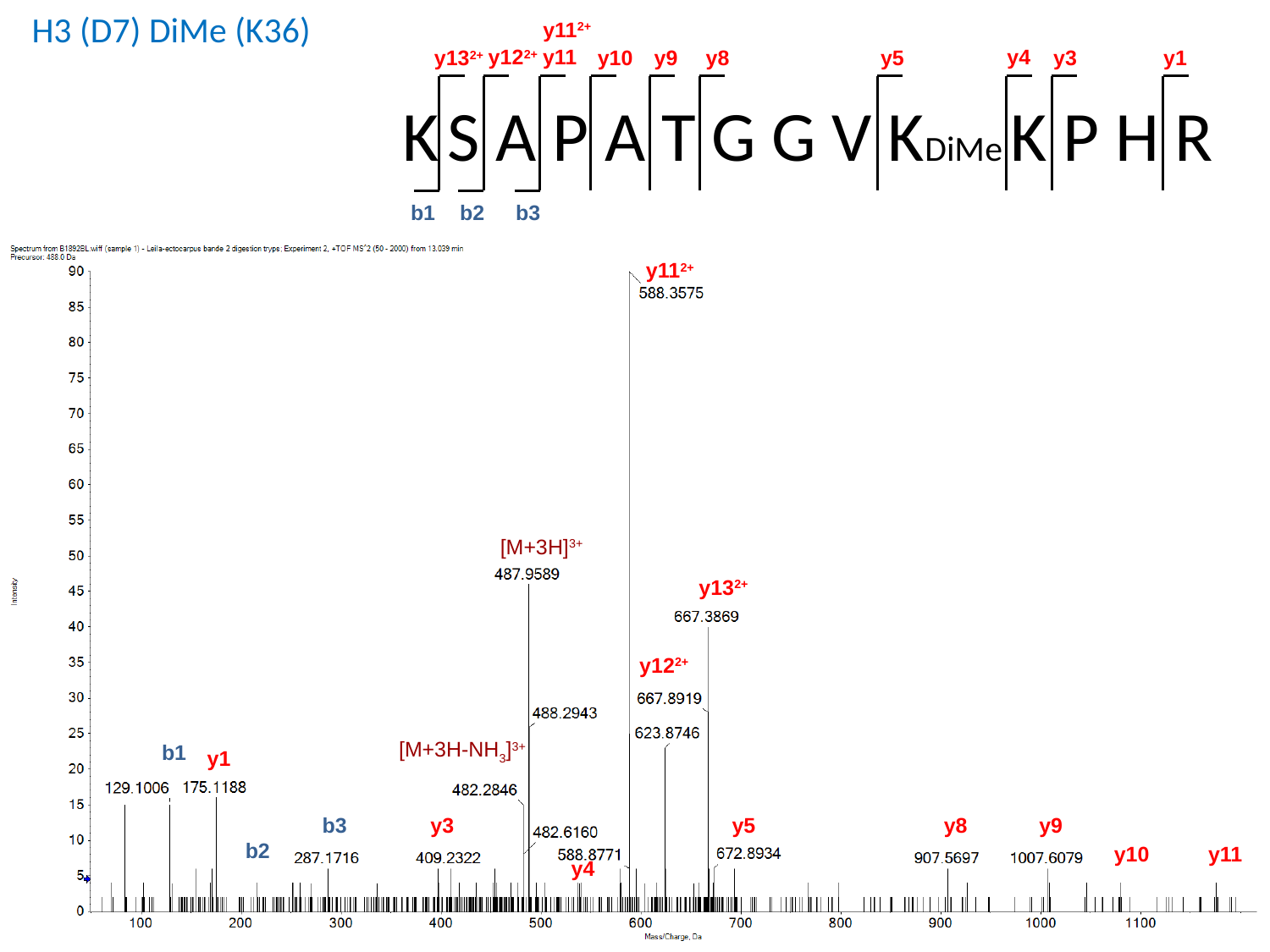

H3 (D7) DiMe (K36)
y112+
y11
y4
y122+
y9
y3
y1
y132+
y10
y8
y5
K S A P A T G G V KDiMe K P H R
b2
b1
b3
y112+
[M+3H]3+
y132+
y122+
[M+3H-NH3]3+
b1
y1
b3
y9
y8
y5
y3
b2
y10
y11
y4

## Slide 15
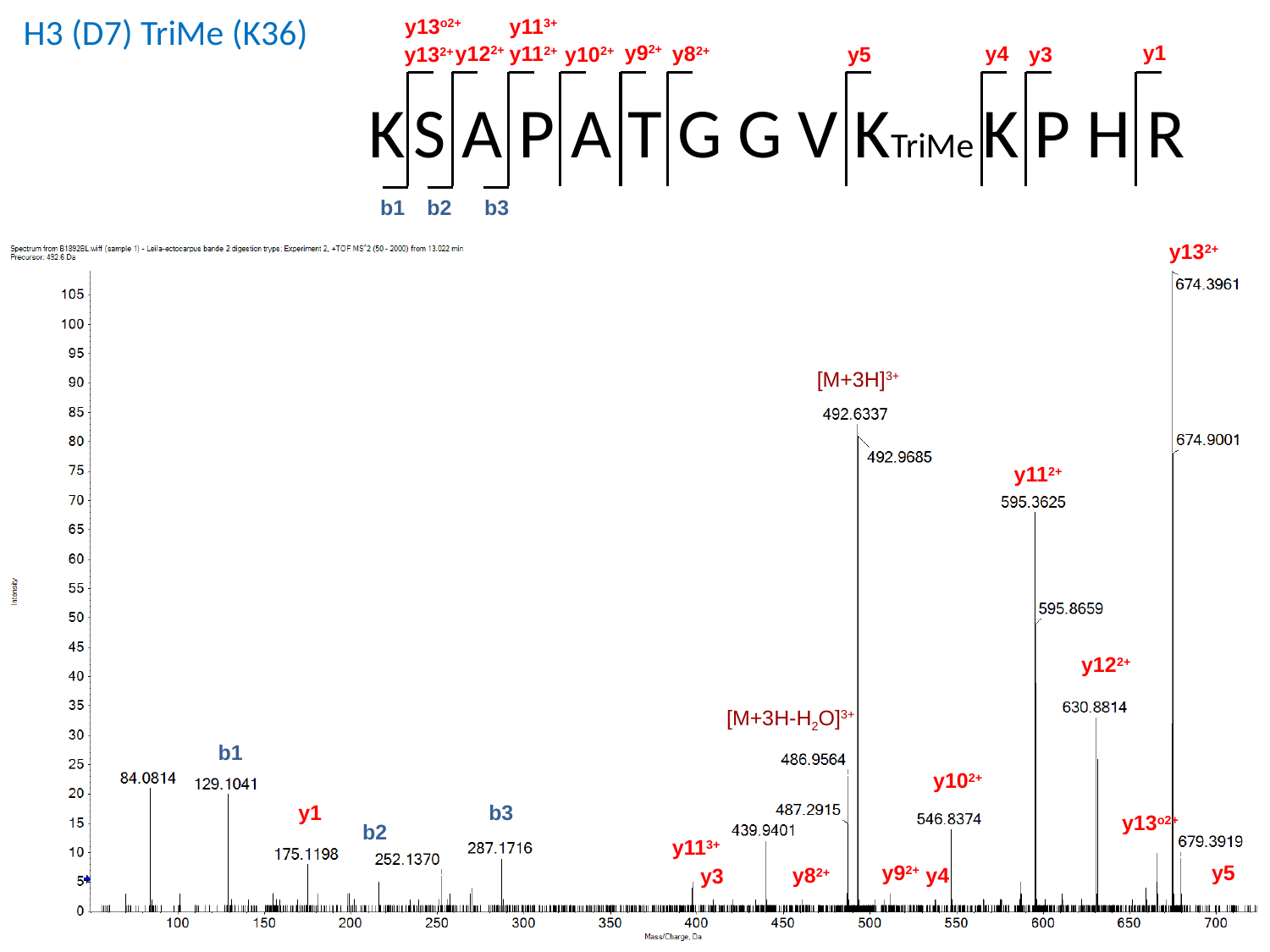

H3 (D7) TriMe (K36)
y13o2+
y113+
y92+
y1
y122+
y4
y82+
y112+
y102+
y5
y3
y132+
K S A P A T G G V KTriMe K P H R
b2
b3
b1
y132+
[M+3H]3+
y112+
y122+
[M+3H-H2O]3+
b1
y102+
b3
y1
y13o2+
b2
y113+
y92+
y5
y4
y82+
y3

## Slide 16
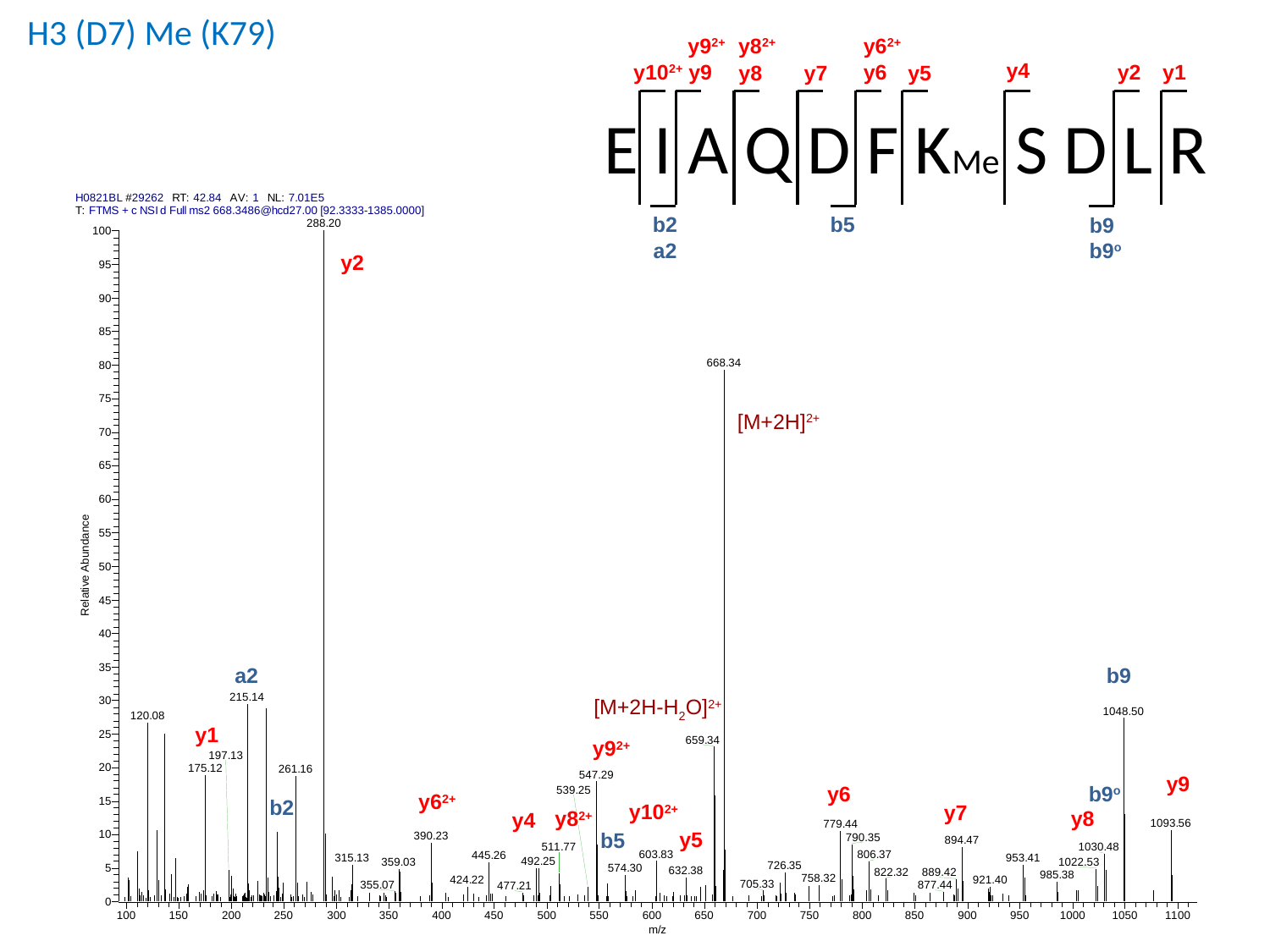

H3 (D7) Me (K79)
y92+
y82+
y62+
y4
y102+
y9
y1
y2
y6
y7
y5
y8
E I A Q D F KMe S D L R
b2
b5
b9
a2
b9o
y2
[M+2H]2+
b9
a2
[M+2H-H2O]2+
y1
y92+
y9
b9o
y6
y62+
b2
y102+
y7
y82+
y8
y4
y5
b5

## Slide 17
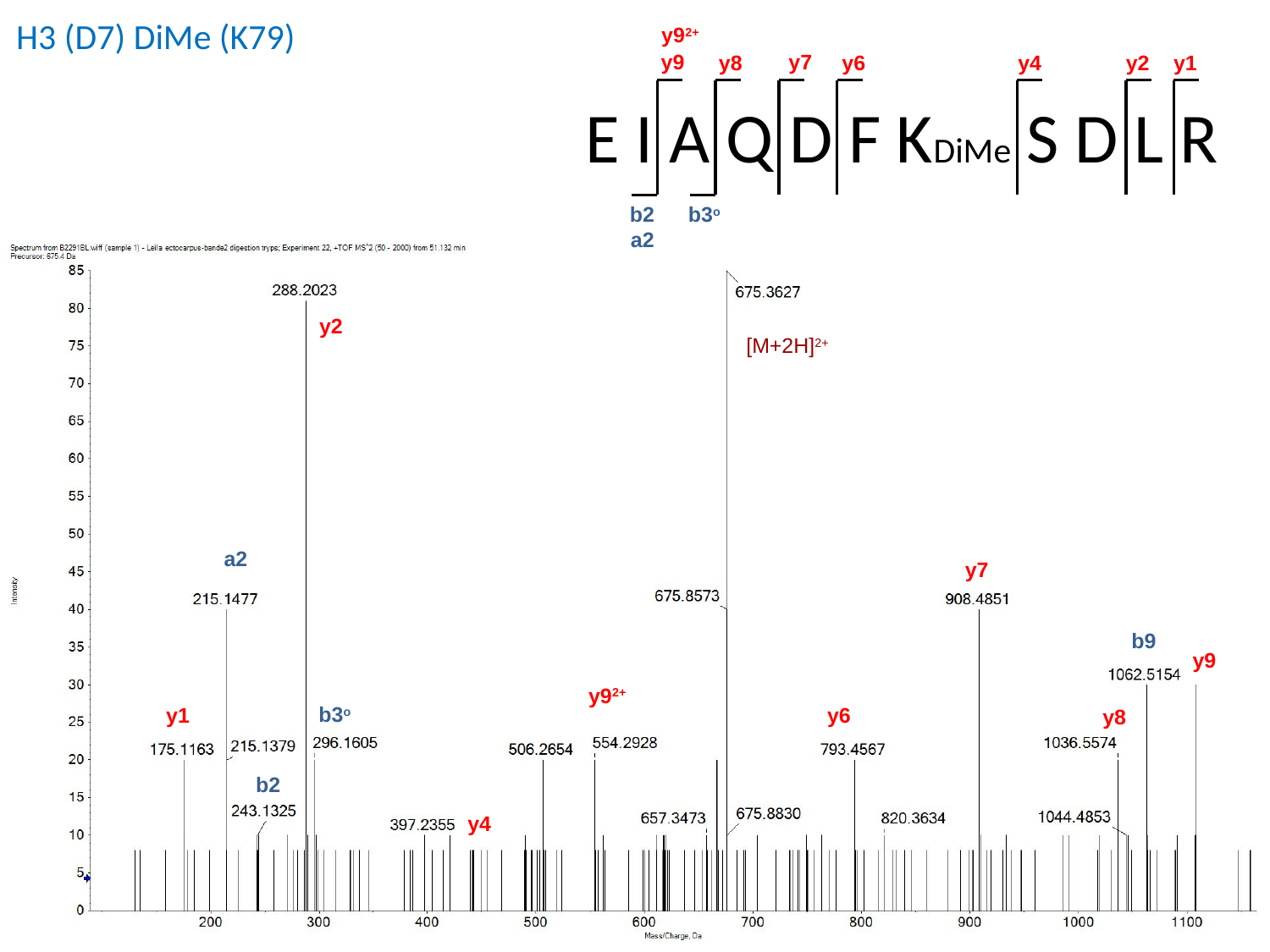

H3 (D7) DiMe (K79)
y92+
y9
y7
y6
y1
y2
y4
y8
E I A Q D F KDiMe S D L R
b2
b3o
a2
y2
[M+2H]2+
a2
y7
b9
y9
y92+
b3o
y6
y1
y8
b2
y4

## Slide 18
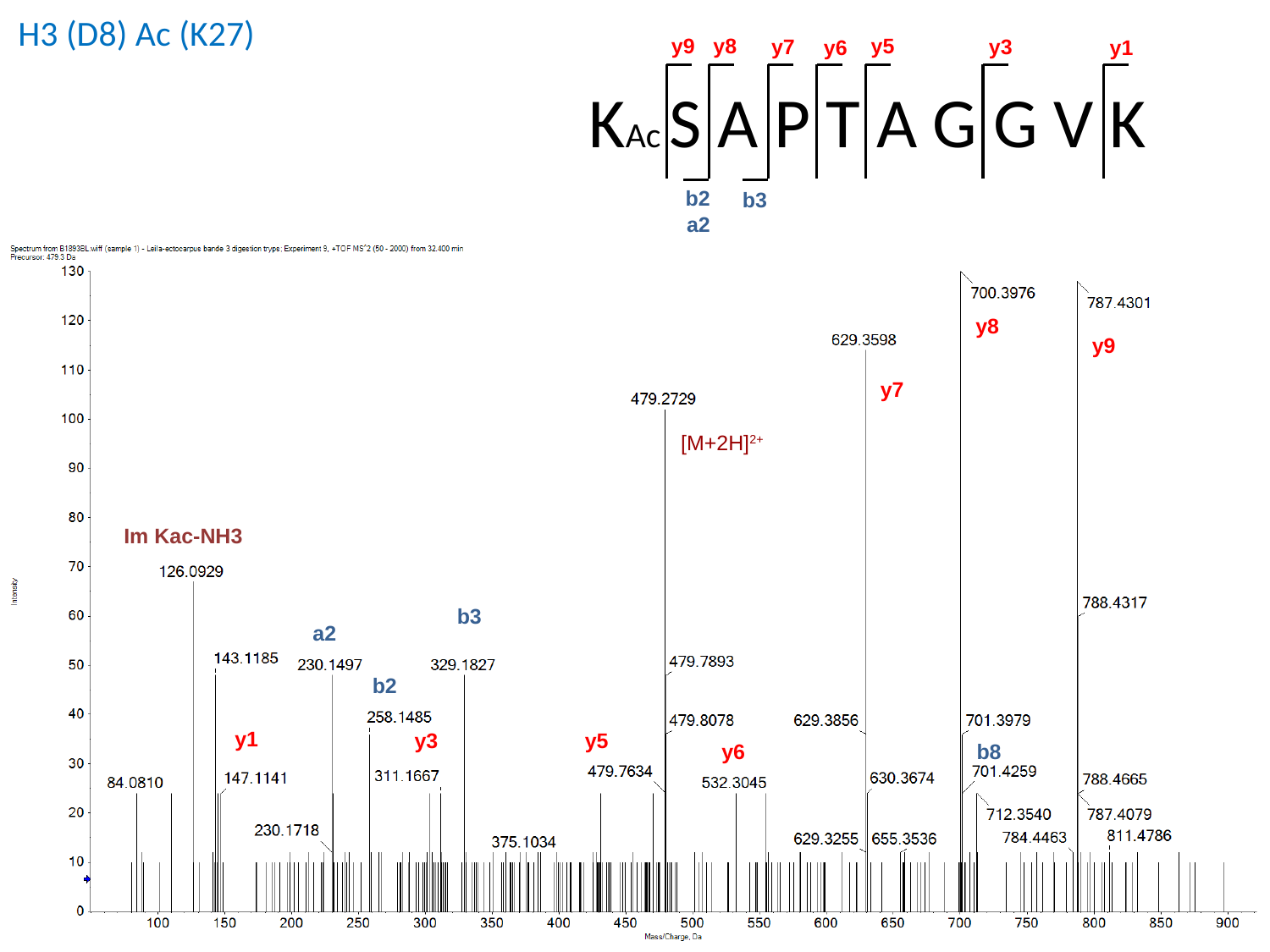

H3 (D8) Ac (K27)
y8
y5
y9
y7
y3
y6
y1
KAc S A P T A G G V K
b2
b3
a2
y8
y9
y7
[M+2H]2+
Im Kac-NH3
b3
a2
b2
y1
y5
y3
b8
y6

## Slide 19
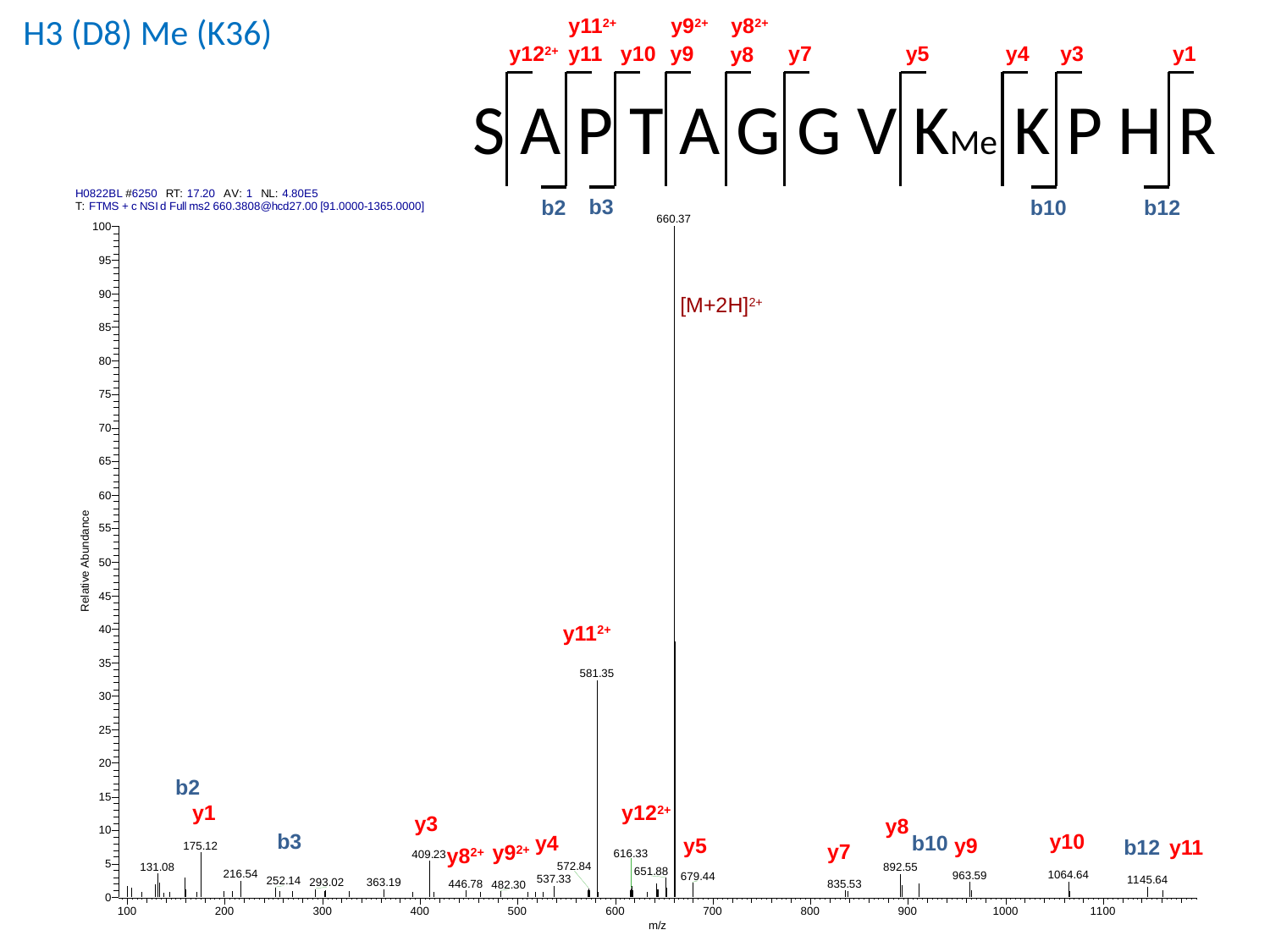

H3 (D8) Me (K36)
y112+
y92+
y82+
y11
y10
y7
y3
y1
y5
y4
y122+
y9
y8
S A P T A G G V KMe K P H R
b3
b10
b2
b12
[M+2H]2+
y112+
b2
y1
y122+
y3
y8
b3
y10
y4
b10
y5
y9
b12
y11
y7
y92+
y82+

## Slide 20
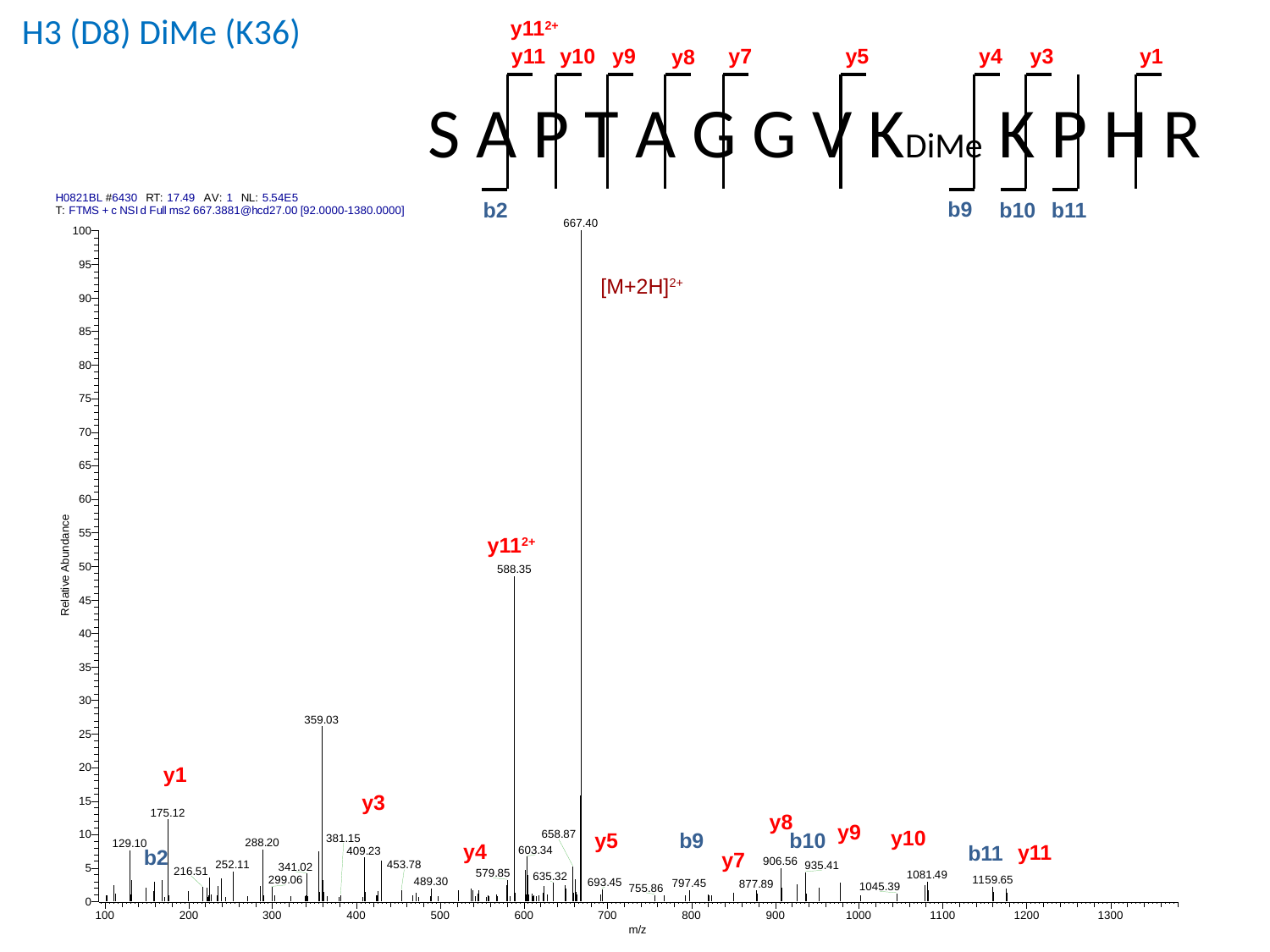

H3 (D8) DiMe (K36)
y112+
y11
y4
y3
y10
y7
y5
y9
y1
y8
S A P T A G G V KDiMe K P H R
b9
b2
b10
b11
[M+2H]2+
y112+
y1
y3
y8
y9
y10
b9
y5
b10
y4
y11
b11
b2
y7

## Slide 21
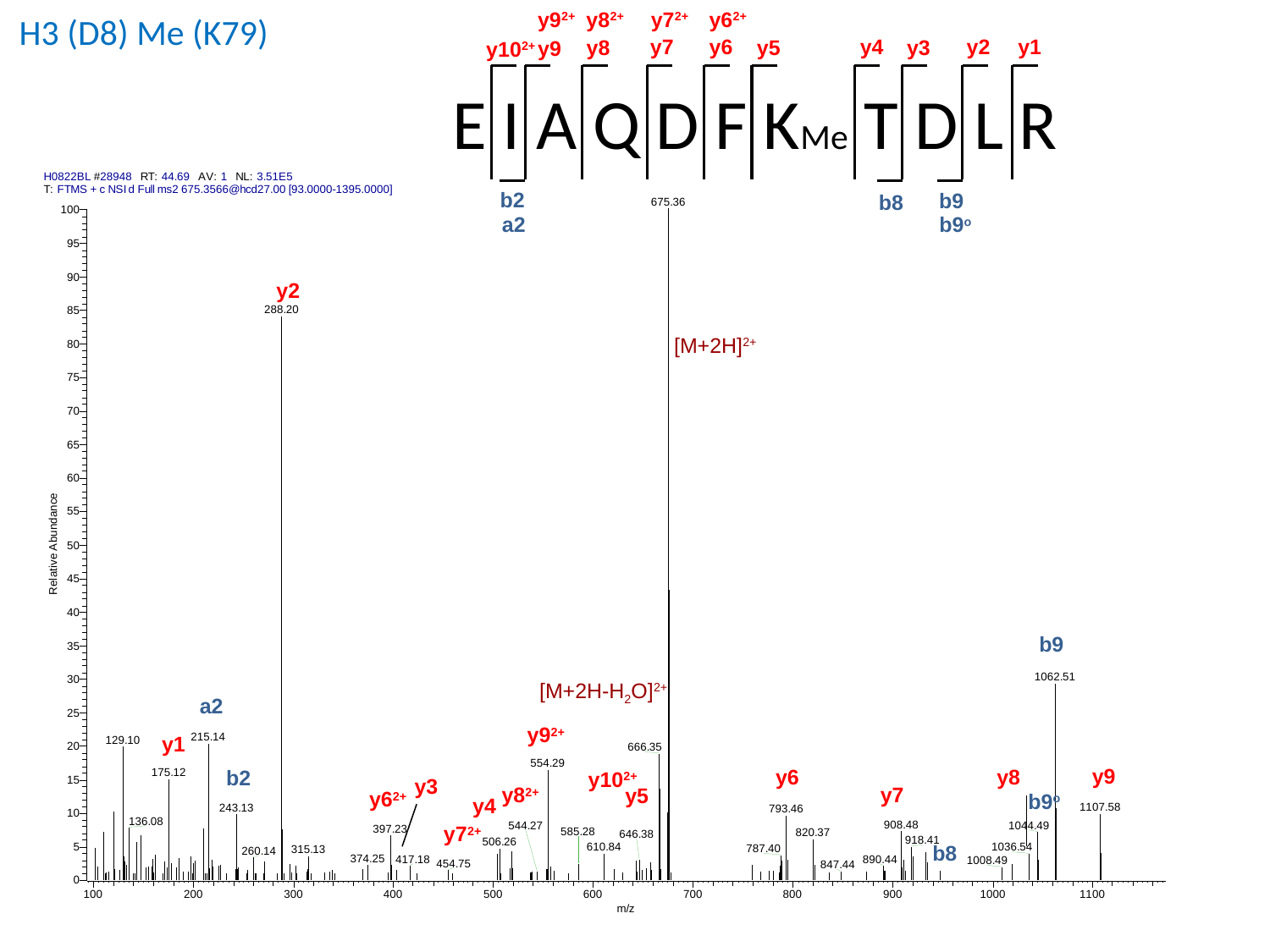

y92+
y82+
y72+
y62+
y7
y6
y4
y1
y2
y8
y5
y3
y9
y102+
E I A Q D F KMe T D L R
b2
b9
b8
a2
b9o
H3 (D8) Me (K79)
y2
[M+2H]2+
b9
[M+2H-H2O]2+
a2
y92+
y1
y9
y8
y6
b2
y102+
y3
y82+
y7
y5
y62+
b9o
y4
y72+
b8

## Slide 22
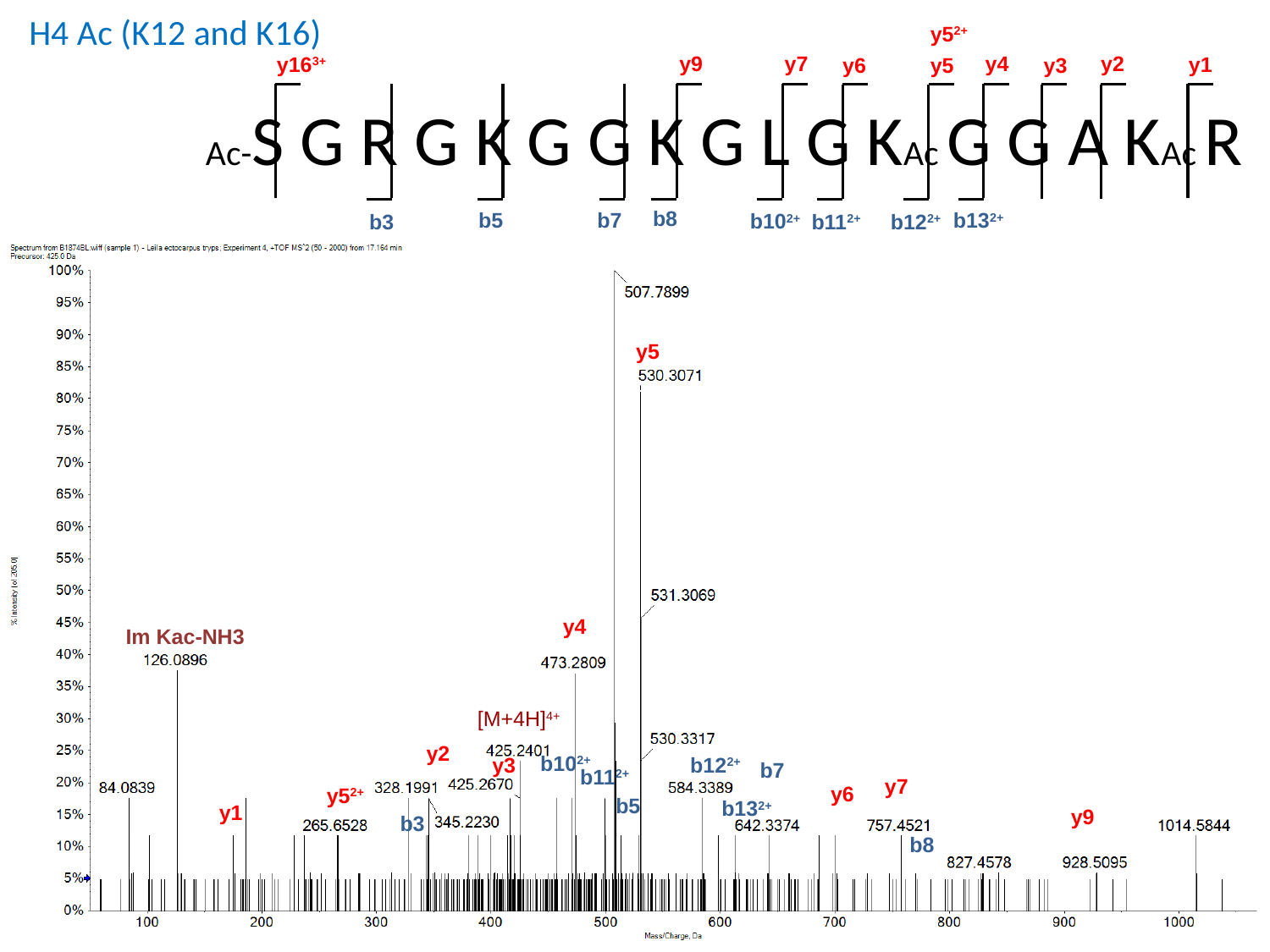

H4 Ac (K12 and K16)
y52+
y4
y2
y7
y9
y163+
y1
y6
y5
y3
Ac-S G R G K G G K G L G KAc G G A KAc R
b8
b7
b5
b132+
b102+
b3
b112+
b122+
y5
y4
[M+4H]4+
y2
b102+
y3
b122+
b7
b112+
y7
y6
y52+
b5
b132+
y1
y9
b3
b8
Im Kac-NH3

## Slide 23
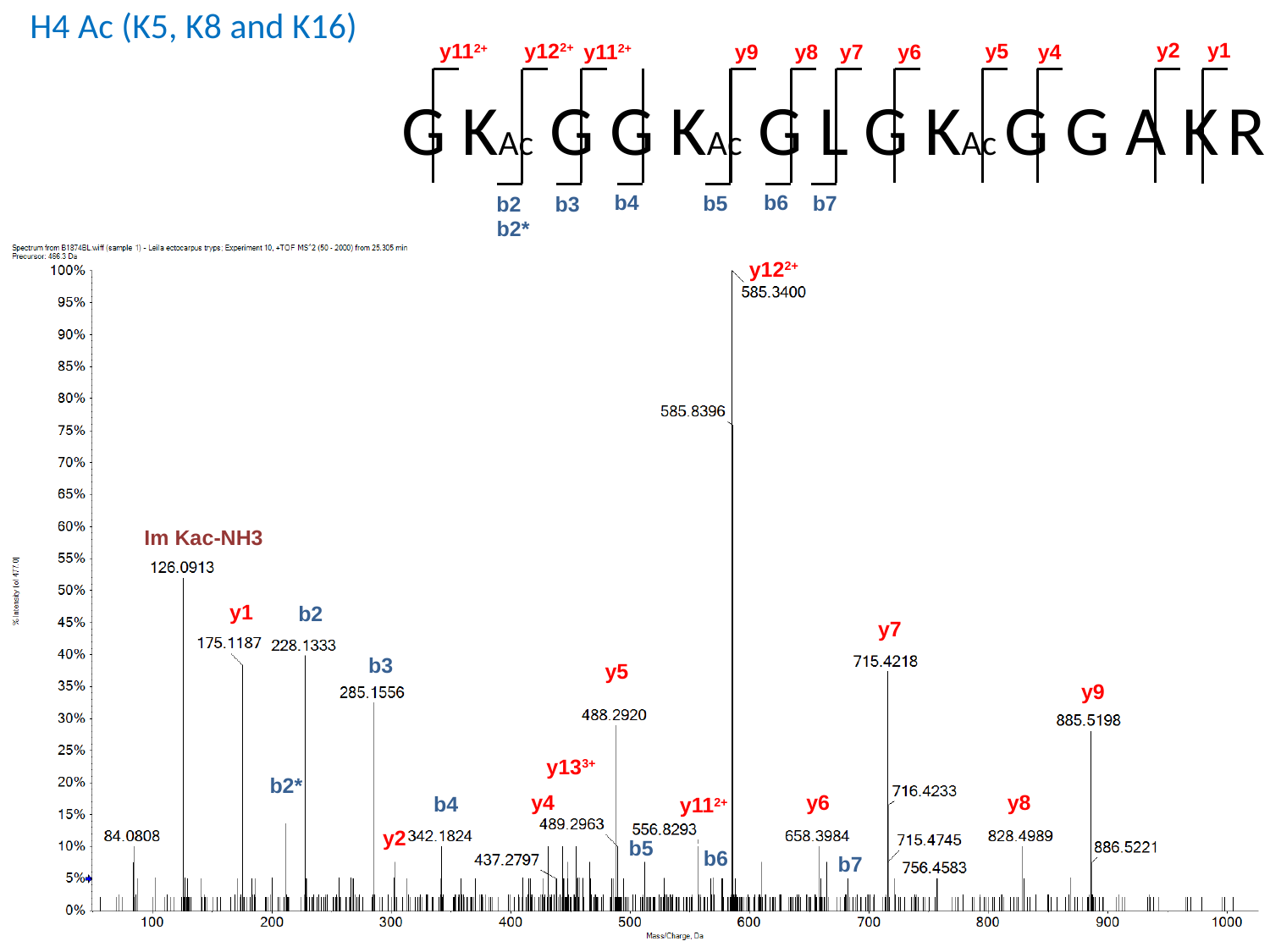

H4 Ac (K5, K8 and K16)
y2
y1
y122+
y112+
y5
y112+
y4
y9
y8
y7
y6
G KAc G G KAc G L G KAc G G A K R
b6
b4
b7
b5
b2
b3
b2*
y122+
Im Kac-NH3
y1
b2
y7
b3
y5
y9
y133+
b2*
y6
y4
y8
b4
y112+
y2
b5
b6
b7

## Slide 24
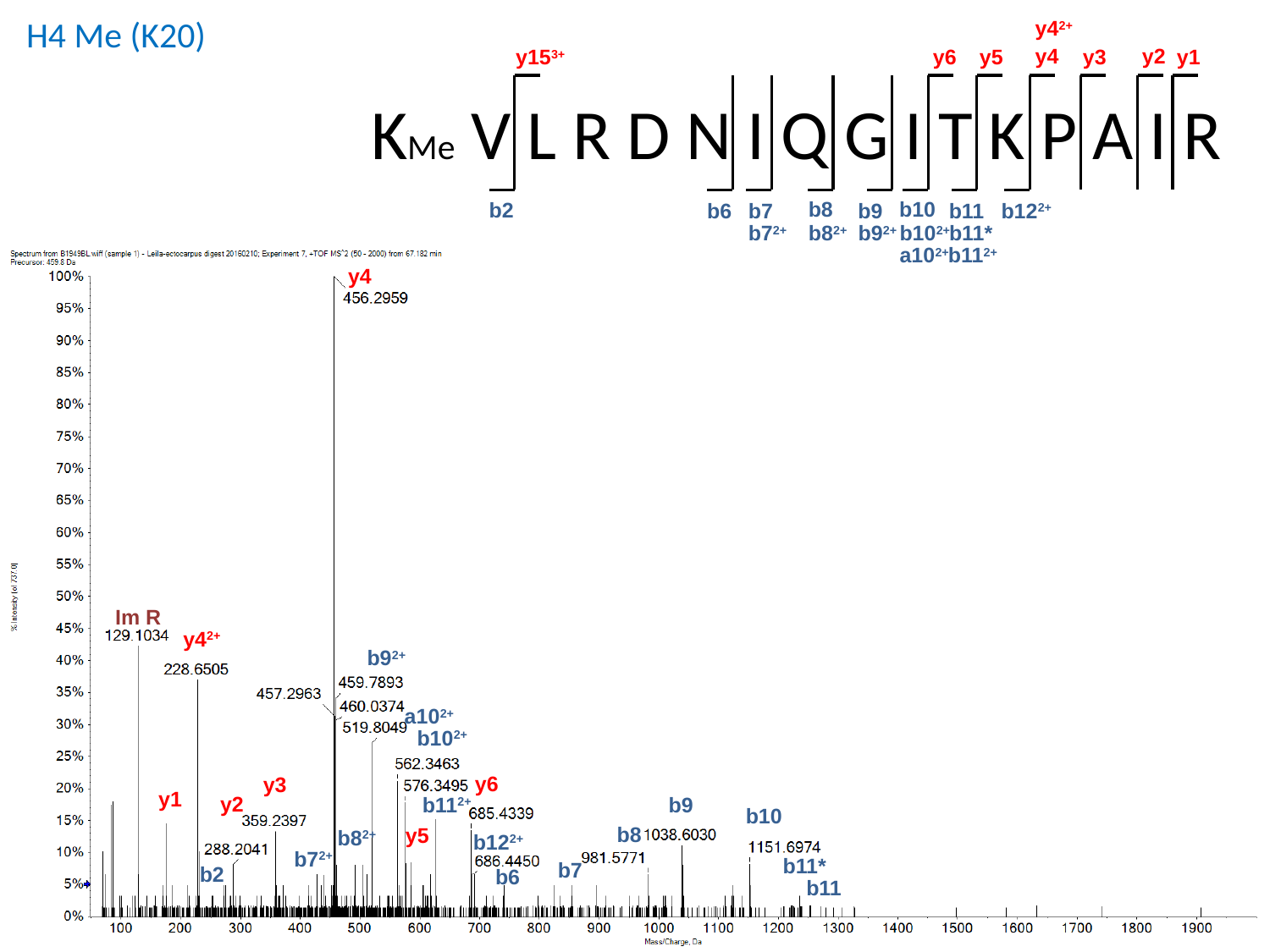

H4 Me (K20)
y42+
y4
y2
y1
y5
y153+
y6
y3
KMe V L R D N I Q G I T K P A I R
b10
b8
b2
b122+
b11
b9
b7
b6
b72+
b82+
b92+
b102+
b11*
a102+
b112+
y4
y42+
b92+
a102+
b102+
y6
y3
y1
y2
b112+
b9
b10
b8
y5
b82+
b122+
b72+
b11*
b7
b2
b6
b11
Im R

## Slide 25
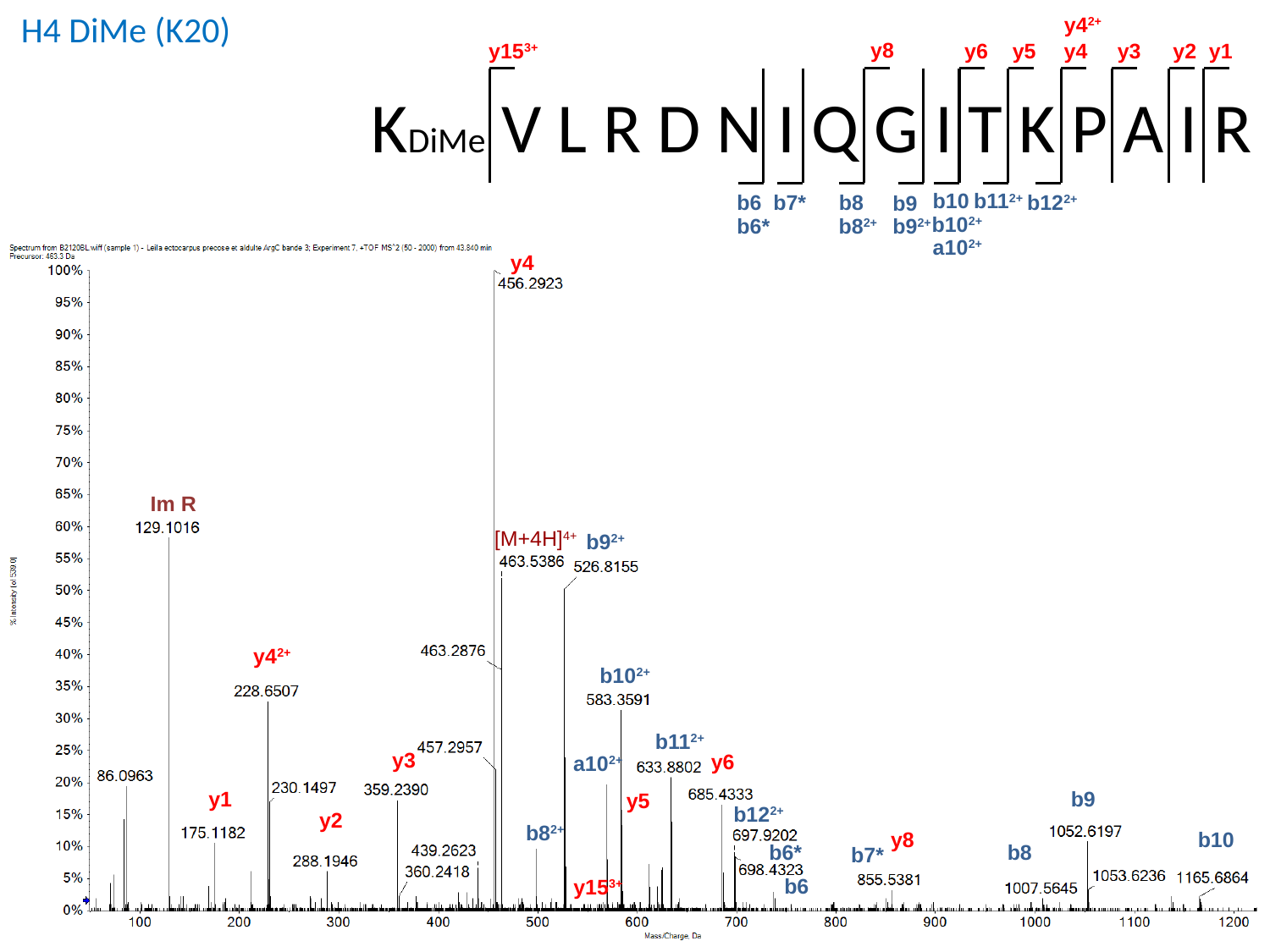

H4 DiMe (K20)
y42+
y8
y153+
y2
y1
y6
y3
y5
y4
KDiMe V L R D N I Q G I T K P A I R
b10
b112+
b7*
b8
b122+
b6
b9
b102+
b6*
b82+
b92+
a102+
[M+4H]4+
b92+
y42+
b102+
b112+
y3
y6
a102+
b9
y1
y5
b122+
y2
b82+
y8
b10
b8
b6*
b7*
b6
y153+
y4
Im R

## Slide 26
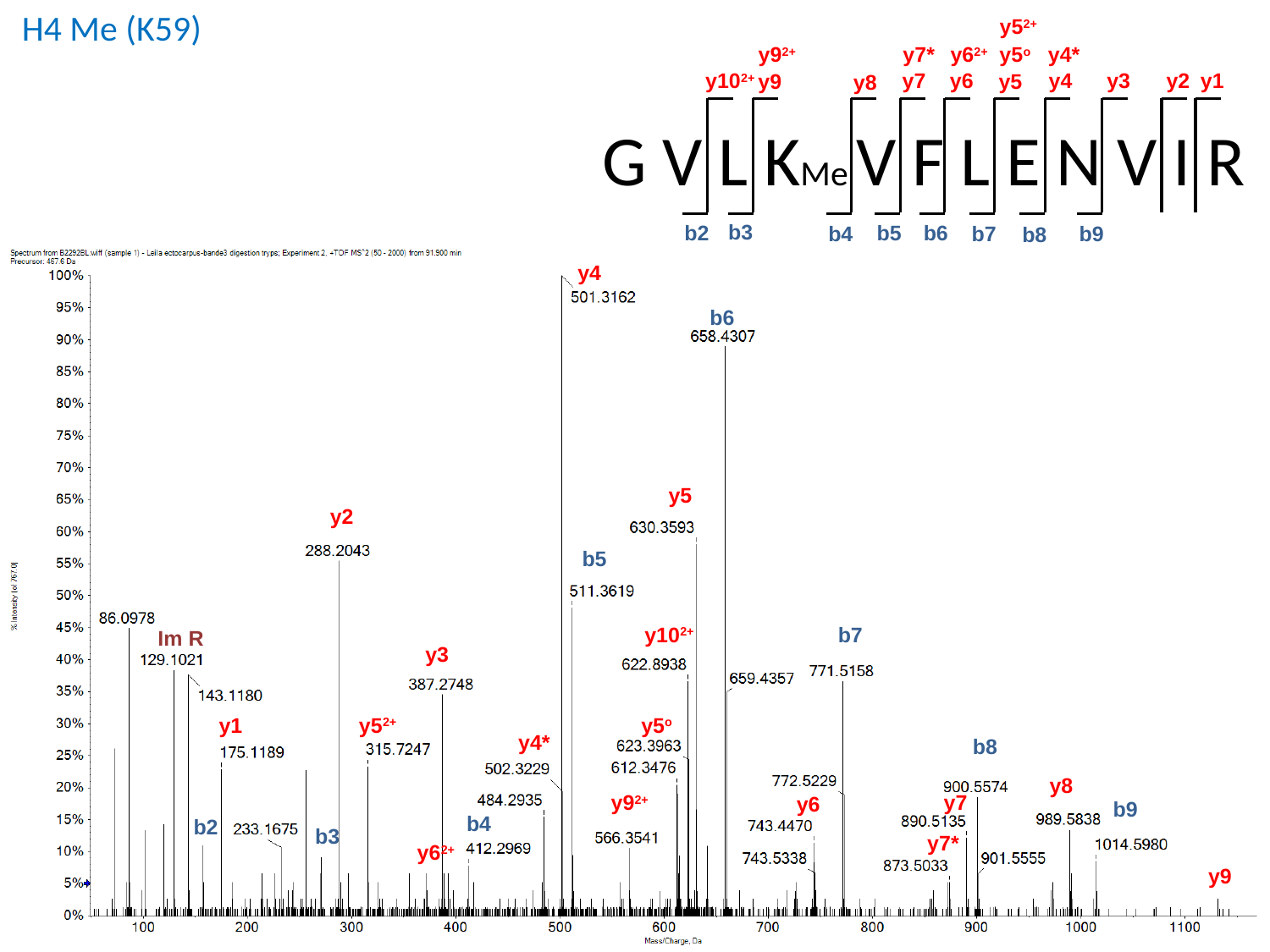

H4 Me (K59)
y52+
y92+
y7*
y62+
y5o
y4*
y1
y102+
y6
y3
y4
y2
y7
y9
y5
y8
G V L KMe V F L E N V I R
b3
b2
b6
b5
b4
b9
b7
b8
y4
b6
y5
y2
b5
y102+
b7
y3
y5o
y1
y52+
y4*
b8
y8
y7
y92+
y6
b9
b4
b2
b3
y7*
y62+
y9
Im R

## Slide 27
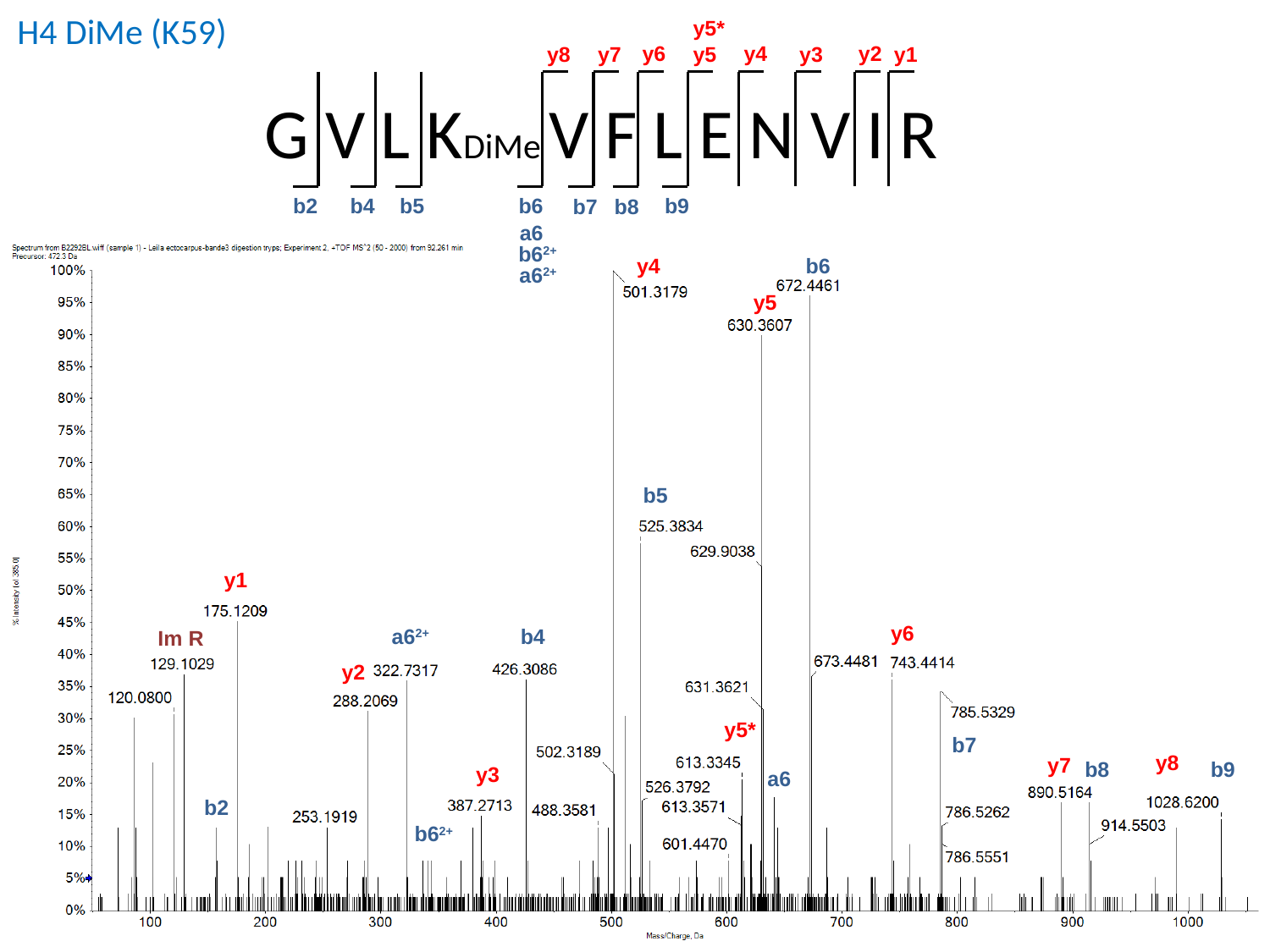

H4 DiMe (K59)
y5*
y4
y2
y6
y8
y5
y1
y7
y3
G V L KDiMe V F L E N V I R
b2
b9
b4
b5
b6
b7
b8
a6
b62+
a62+
b6
y5
b5
y1
y6
a62+
b4
y2
y5*
b7
y8
y7
b9
b8
y3
a6
b2
b62+
y4
Im R

## Slide 28
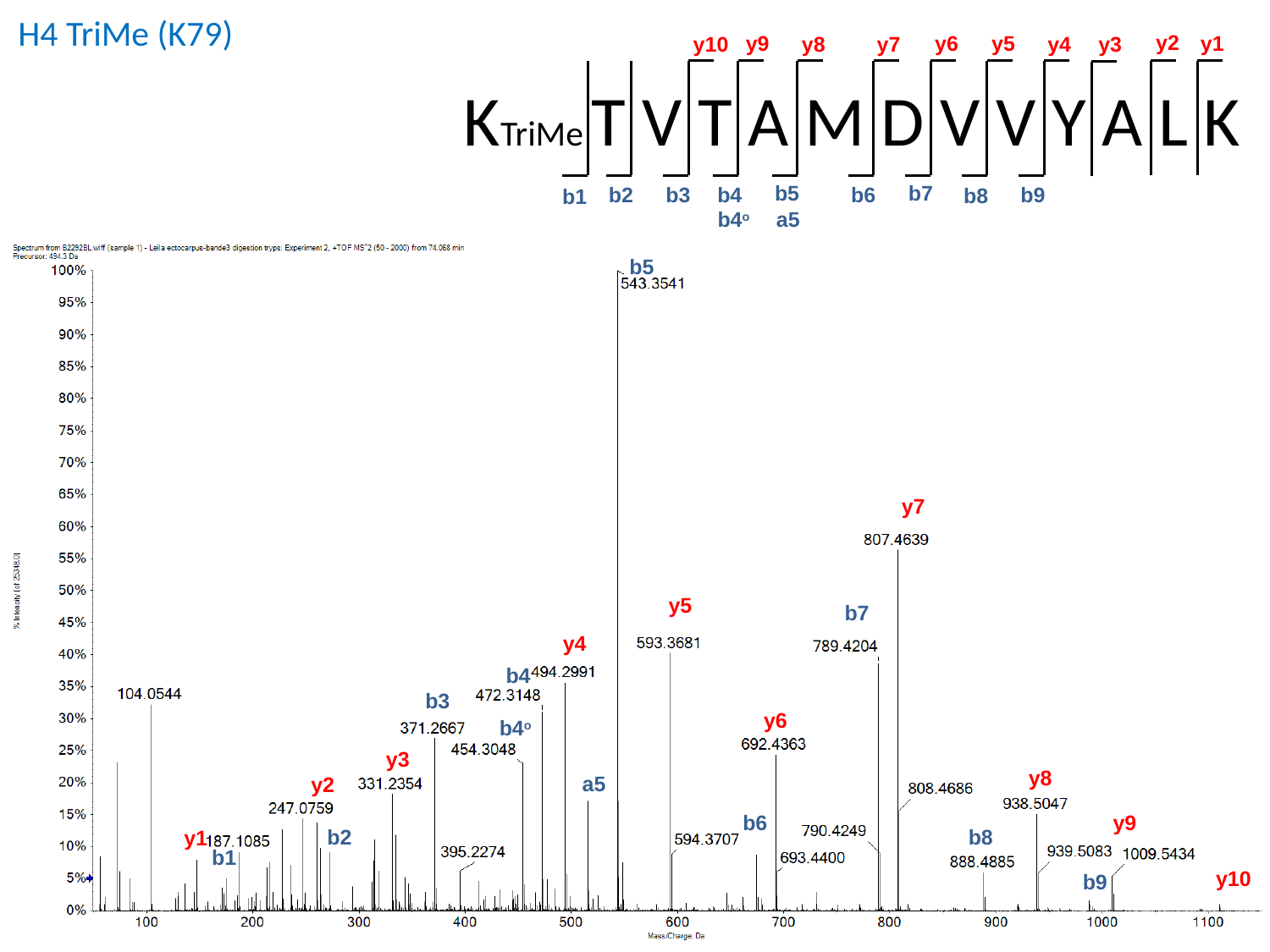

H4 TriMe (K79)
y2
y6
y5
y9
y1
y10
y4
y8
y7
y3
KTriMe T V T A M D V V Y A L K
b5
b7
b2
b6
b9
b3
b4
b8
b1
b4o
a5
b5
y7
y5
b7
y4
b4
b3
y6
b4o
y3
y8
a5
y2
b6
y9
b8
b2
y1
b1
y10
b9

## Slide 29
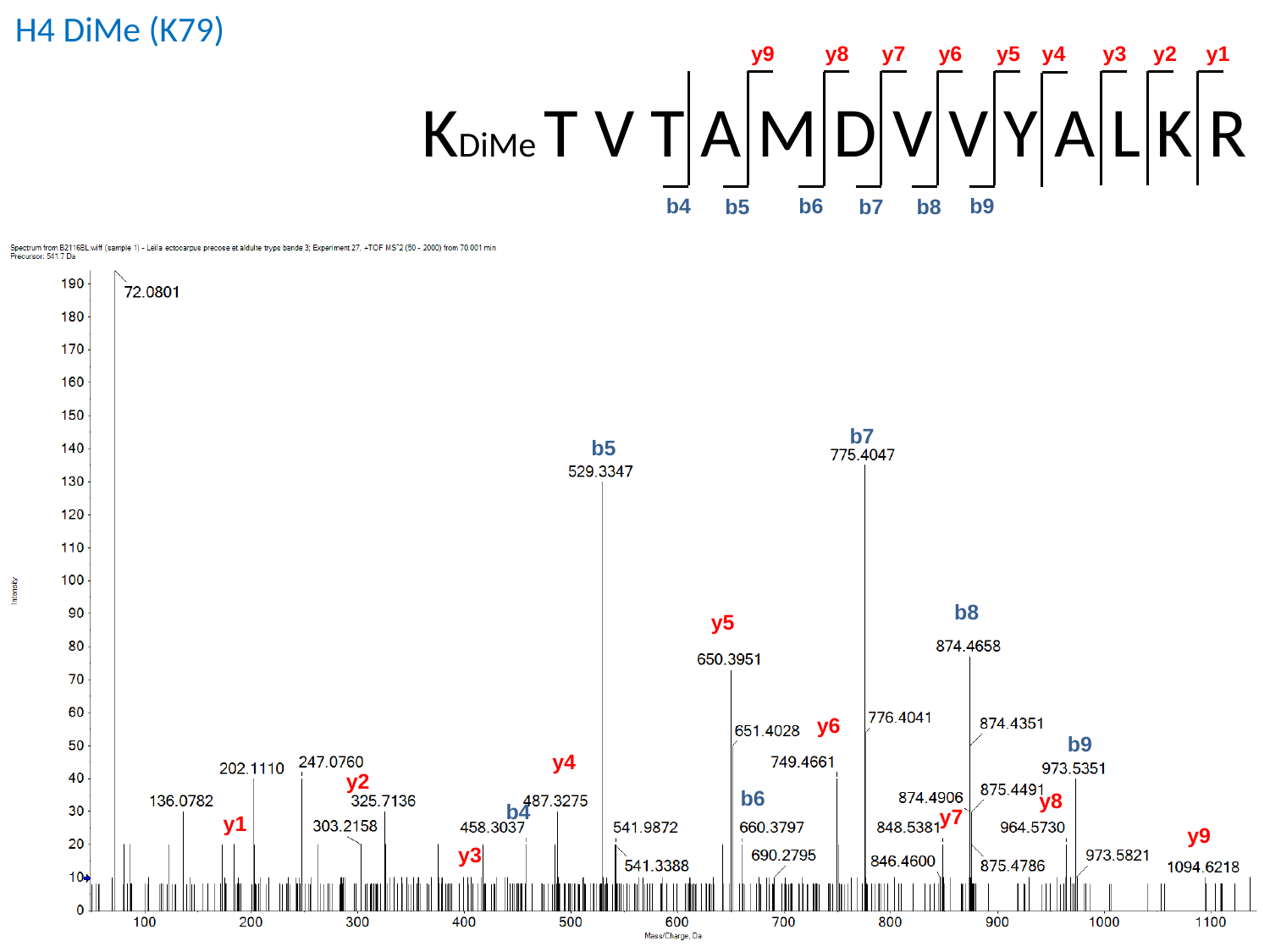

H4 DiMe (K79)
y9
y8
y7
y6
y5
y4
y3
y2
y1
KDiMe T V T A M D V V Y A L K R
b9
b6
b4
b7
b5
b8
b5
b8
y5
y6
b9
y4
y2
b6
y8
b4
y7
y1
y9
y3
b7

## Slide 30
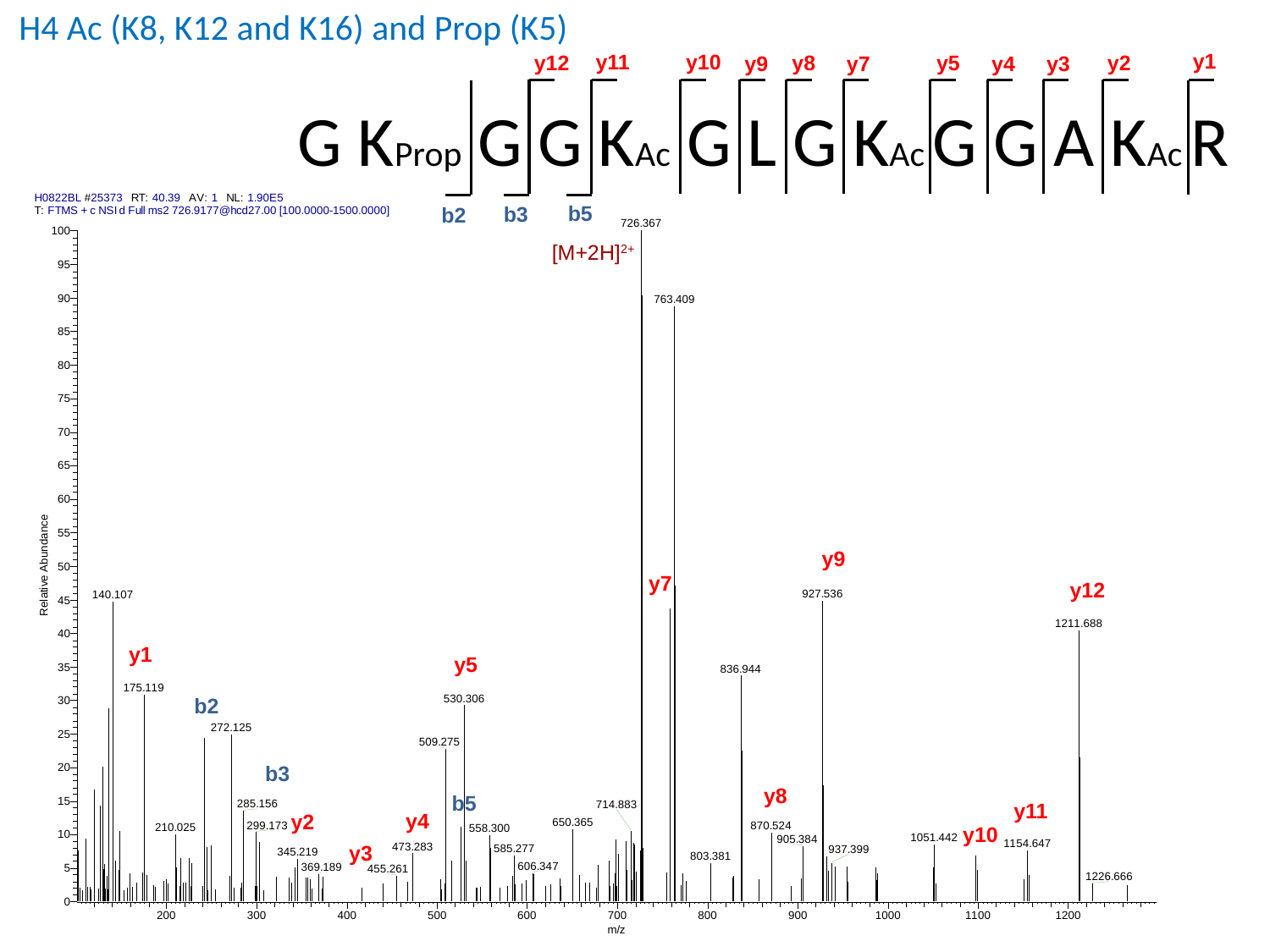

H4 Ac (K8, K12 and K16) and Prop (K5)
y1
y11
y10
y2
y12
y8
y5
y3
y9
y7
y4
G KProp G G KAc G L G KAc G G A KAc R
b5
b3
b2
[M+2H]2+
y9
y7
y12
y1
y5
b2
b3
y8
b5
y11
y4
y2
y10
y3
