# Supplementary material for: Histone modifications during the life cycle of the brown alga Ectocarpus
Source: Genome Biol. 2021 Jan 4;22:12. doi: 10.1186/s13059-020-02216-8 (PMC7784034; doi:10.1186/s13059-020-02216-8)
Supplement: Supplementary file 4 — Additional file 4: Figure S3. Immunoblots of histone PTMs. Figure S4. Genomic distribution of the histone PTMs H3K79me2 and H4K20me3. Figure S5. Boundaries of H3K79me2 regions are preferentially located near TSSs and TESs. Figure S6. Comparison of genes marked with H3K79me2 alone, H4K20me3 alone or both H3K79me2 and H4K20me3. Figure S7. H3K79me2, H4K20me3, H3K36me3, H3K4me2, H3K4me3, H3K9ac, H3K14ac and H3K27ac signals for a region of chromosome 6 spanning an inserted viral genome. Figure S8. Correlations of transcript abundances and histone PTM signals for divergently transcribed pairs of genes. Figure S9. The histone PTM H2AK119ub1 was not detected in Ectocarpus. Figure S10. Pearson correlation scores for comparisons of the genomic distributions of ChIP-seq signal peaks for duplicate assays of the eight histone PTMs during both the sporophyte and gametophyte generations. [file 13059_2020_2216_MOESM4_ESM.pptx]

## Slide 1
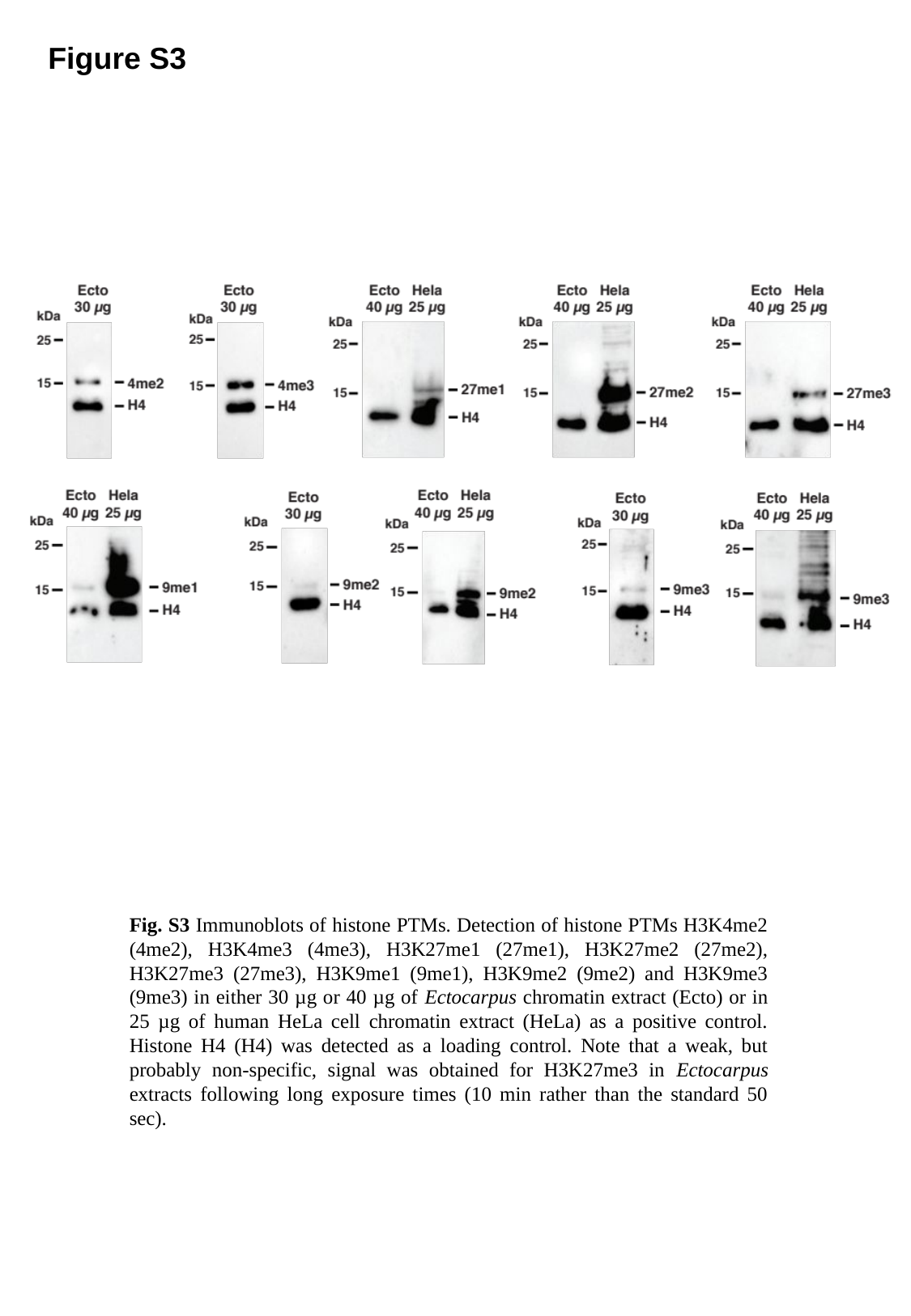

Figure S3
Fig. S3 Immunoblots of histone PTMs. Detection of histone PTMs H3K4me2 (4me2), H3K4me3 (4me3), H3K27me1 (27me1), H3K27me2 (27me2), H3K27me3 (27me3), H3K9me1 (9me1), H3K9me2 (9me2) and H3K9me3 (9me3) in either 30 µg or 40 µg of Ectocarpus chromatin extract (Ecto) or in 25 µg of human HeLa cell chromatin extract (HeLa) as a positive control. Histone H4 (H4) was detected as a loading control. Note that a weak, but probably non-specific, signal was obtained for H3K27me3 in Ectocarpus extracts following long exposure times (10 min rather than the standard 50 sec).

## Slide 2
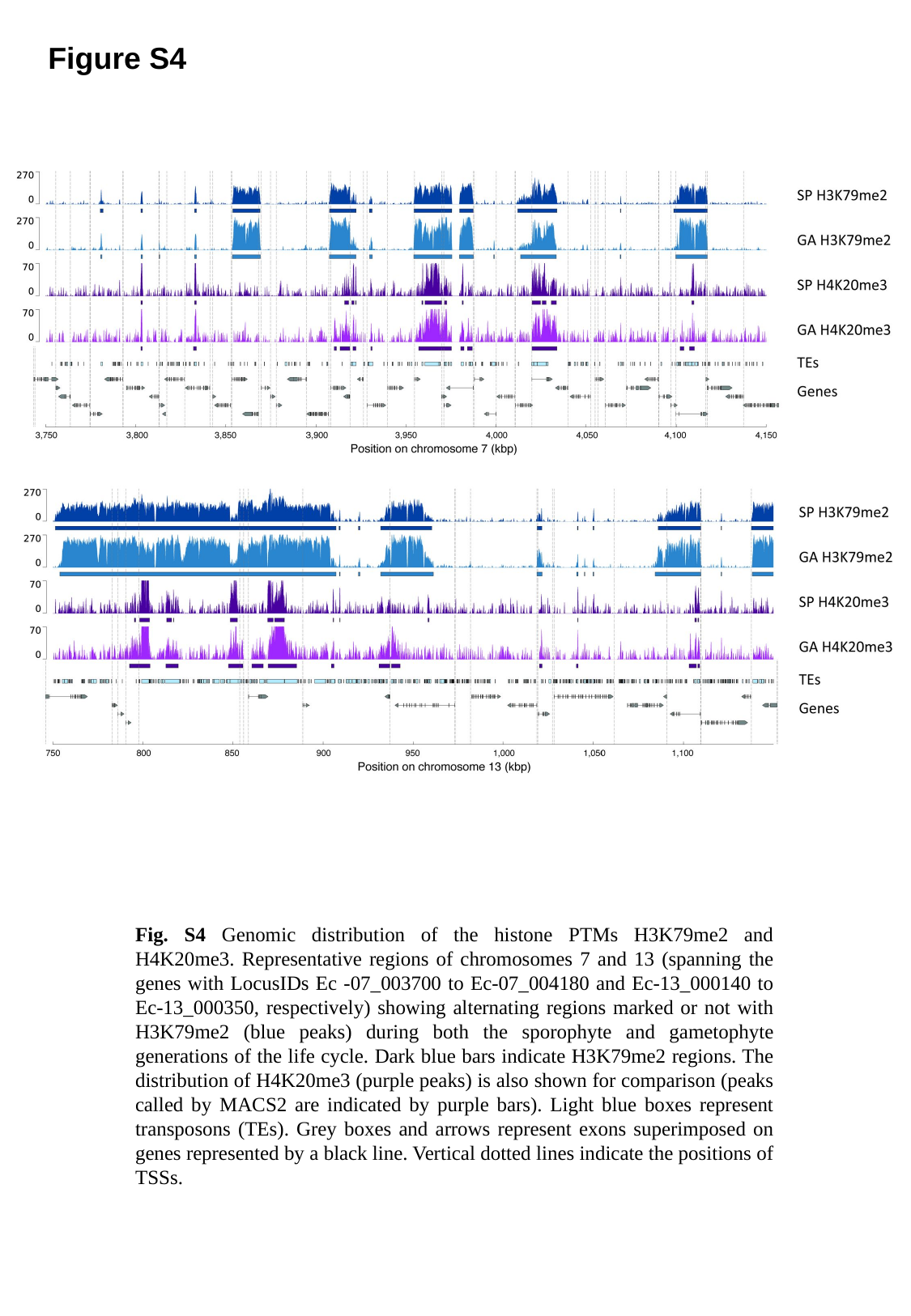

Figure S4
Fig. S4 Genomic distribution of the histone PTMs H3K79me2 and H4K20me3. Representative regions of chromosomes 7 and 13 (spanning the genes with LocusIDs Ec -07_003700 to Ec-07_004180 and Ec-13_000140 to Ec-13_000350, respectively) showing alternating regions marked or not with H3K79me2 (blue peaks) during both the sporophyte and gametophyte generations of the life cycle. Dark blue bars indicate H3K79me2 regions. The distribution of H4K20me3 (purple peaks) is also shown for comparison (peaks called by MACS2 are indicated by purple bars). Light blue boxes represent transposons (TEs). Grey boxes and arrows represent exons superimposed on genes represented by a black line. Vertical dotted lines indicate the positions of TSSs.

## Slide 3
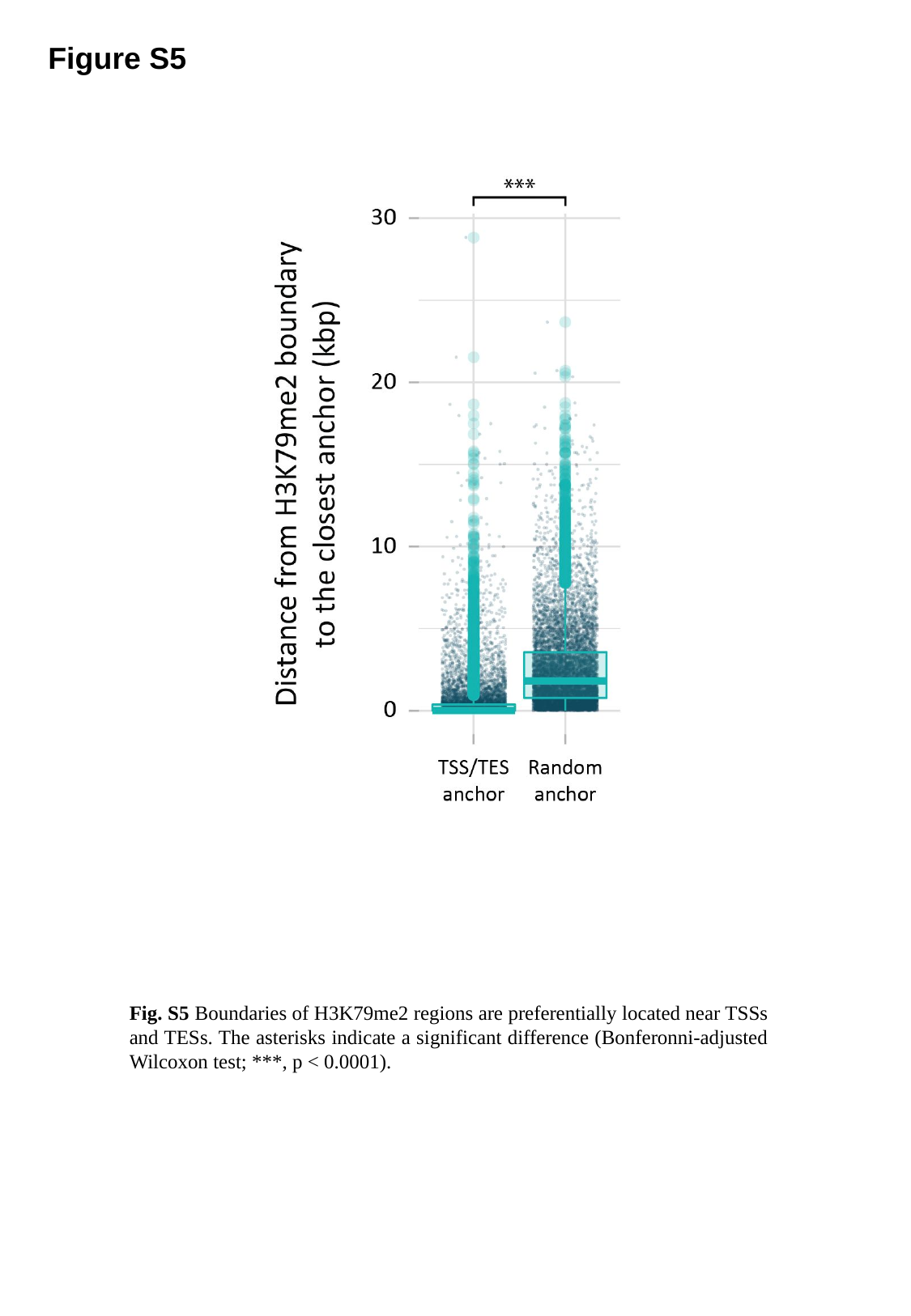

Figure S5
Fig. S5 Boundaries of H3K79me2 regions are preferentially located near TSSs and TESs. The asterisks indicate a significant difference (Bonferonni-adjusted Wilcoxon test; ***, p < 0.0001).

## Slide 4
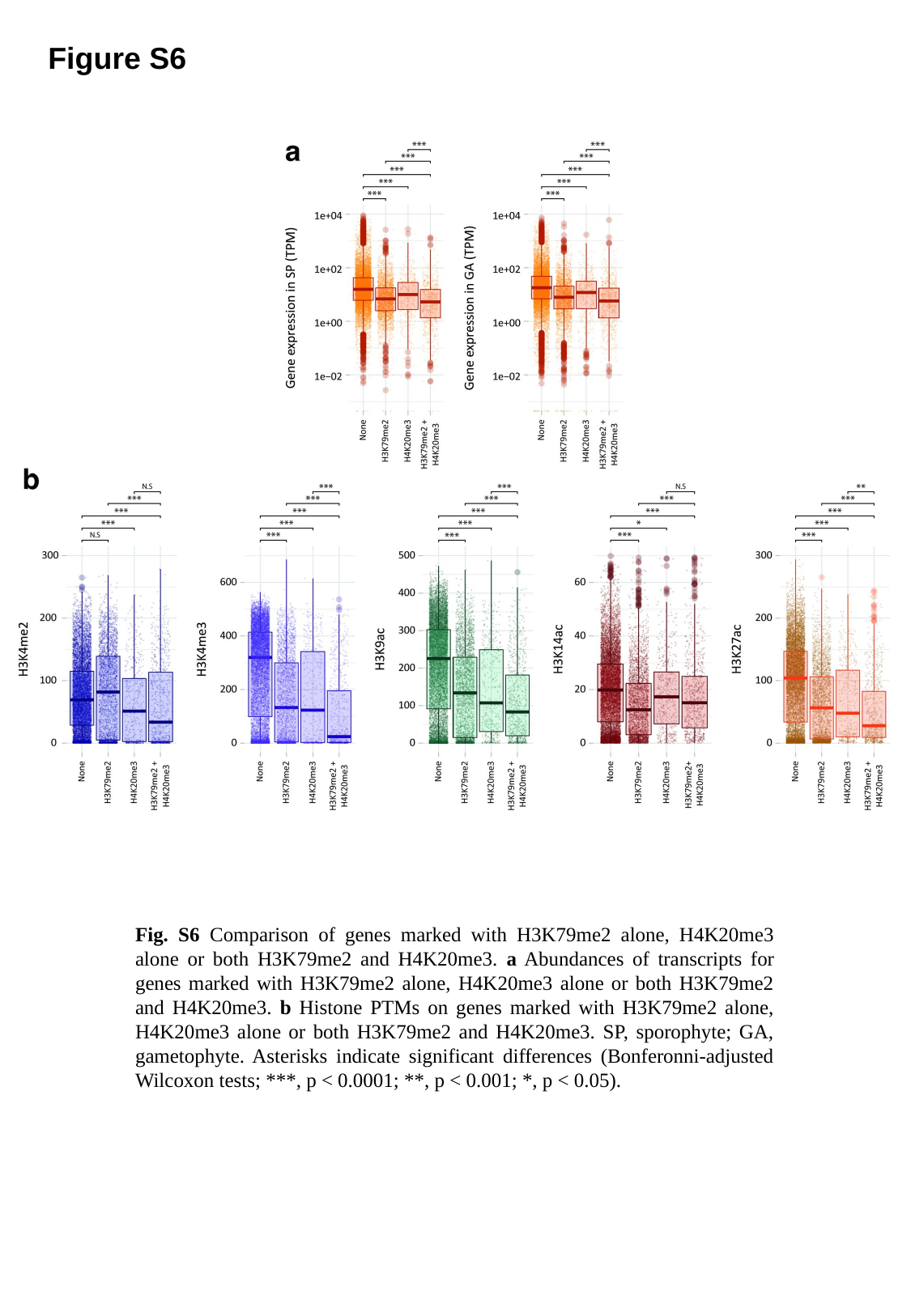

Figure S6
Fig. S6 Comparison of genes marked with H3K79me2 alone, H4K20me3 alone or both H3K79me2 and H4K20me3. a Abundances of transcripts for genes marked with H3K79me2 alone, H4K20me3 alone or both H3K79me2 and H4K20me3. b Histone PTMs on genes marked with H3K79me2 alone, H4K20me3 alone or both H3K79me2 and H4K20me3. SP, sporophyte; GA, gametophyte. Asterisks indicate significant differences (Bonferonni-adjusted Wilcoxon tests; ***, p < 0.0001; **, p < 0.001; *, p < 0.05).

## Slide 5
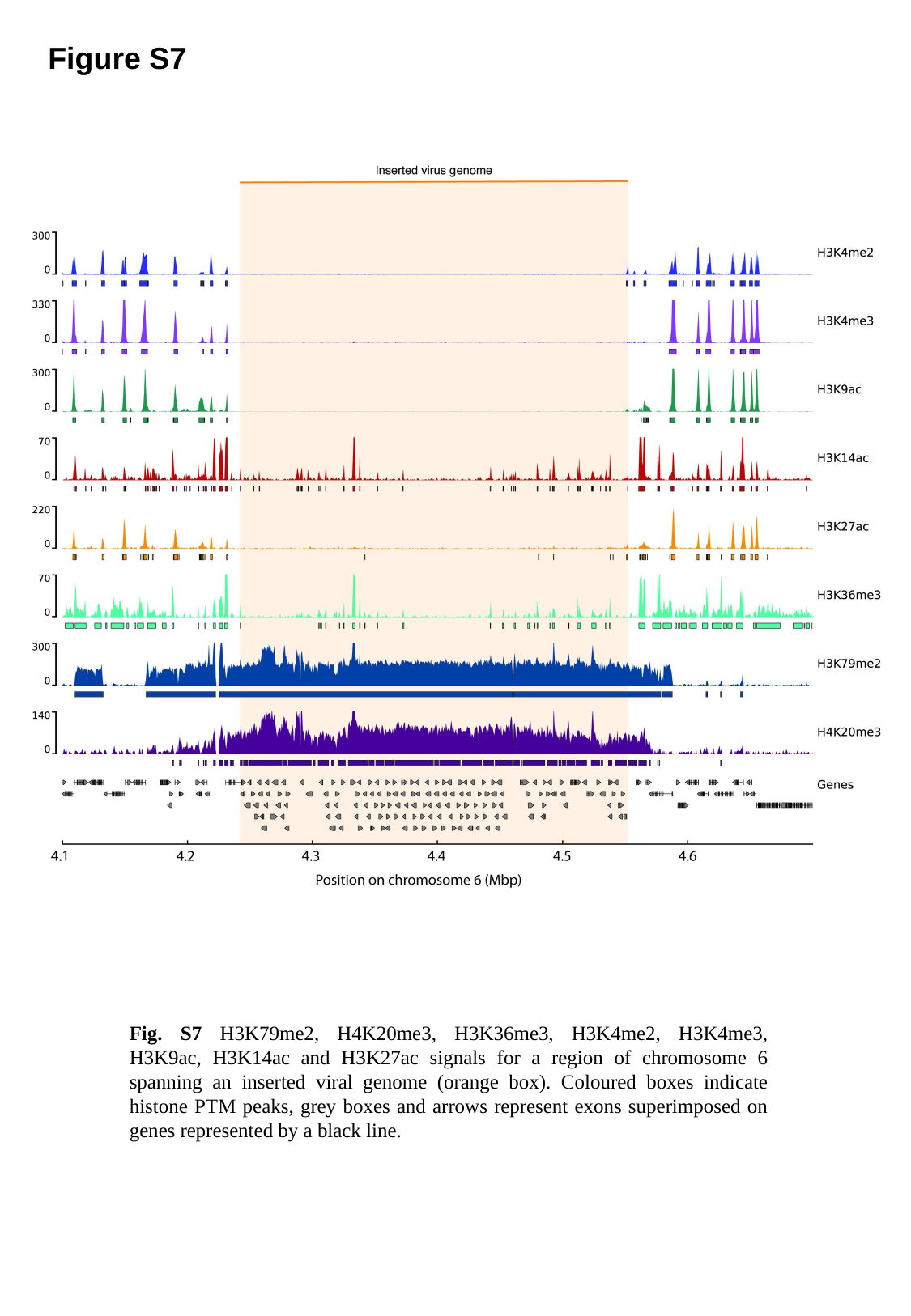

Figure S7
Fig. S7 H3K79me2, H4K20me3, H3K36me3, H3K4me2, H3K4me3, H3K9ac, H3K14ac and H3K27ac signals for a region of chromosome 6 spanning an inserted viral genome (orange box). Coloured boxes indicate histone PTM peaks, grey boxes and arrows represent exons superimposed on genes represented by a black line.

## Slide 6
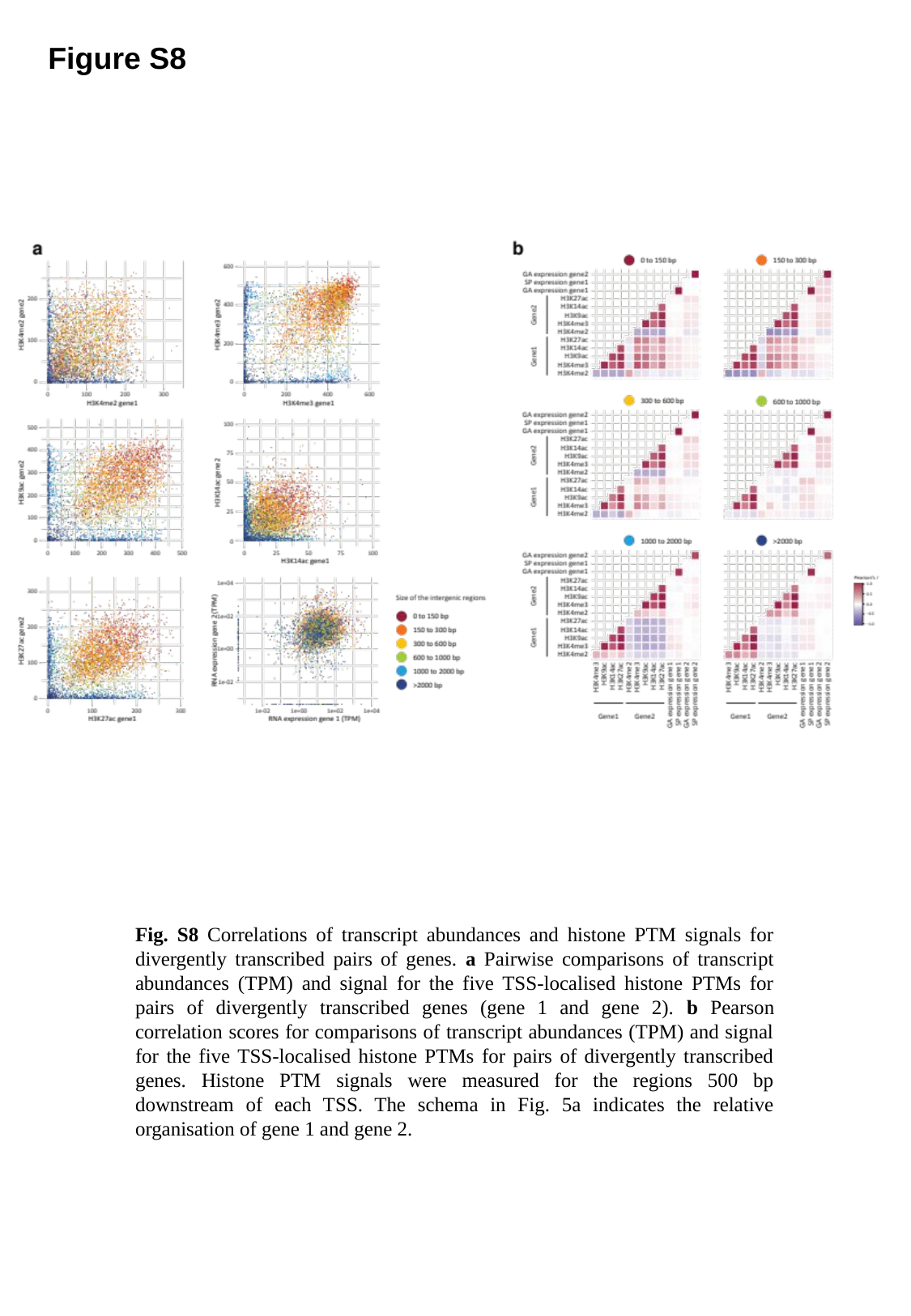

Figure S8
Fig. S8 Correlations of transcript abundances and histone PTM signals for divergently transcribed pairs of genes. a Pairwise comparisons of transcript abundances (TPM) and signal for the five TSS-localised histone PTMs for pairs of divergently transcribed genes (gene 1 and gene 2). b Pearson correlation scores for comparisons of transcript abundances (TPM) and signal for the five TSS-localised histone PTMs for pairs of divergently transcribed genes. Histone PTM signals were measured for the regions 500 bp downstream of each TSS. The schema in Fig. 5a indicates the relative organisation of gene 1 and gene 2.

## Slide 7
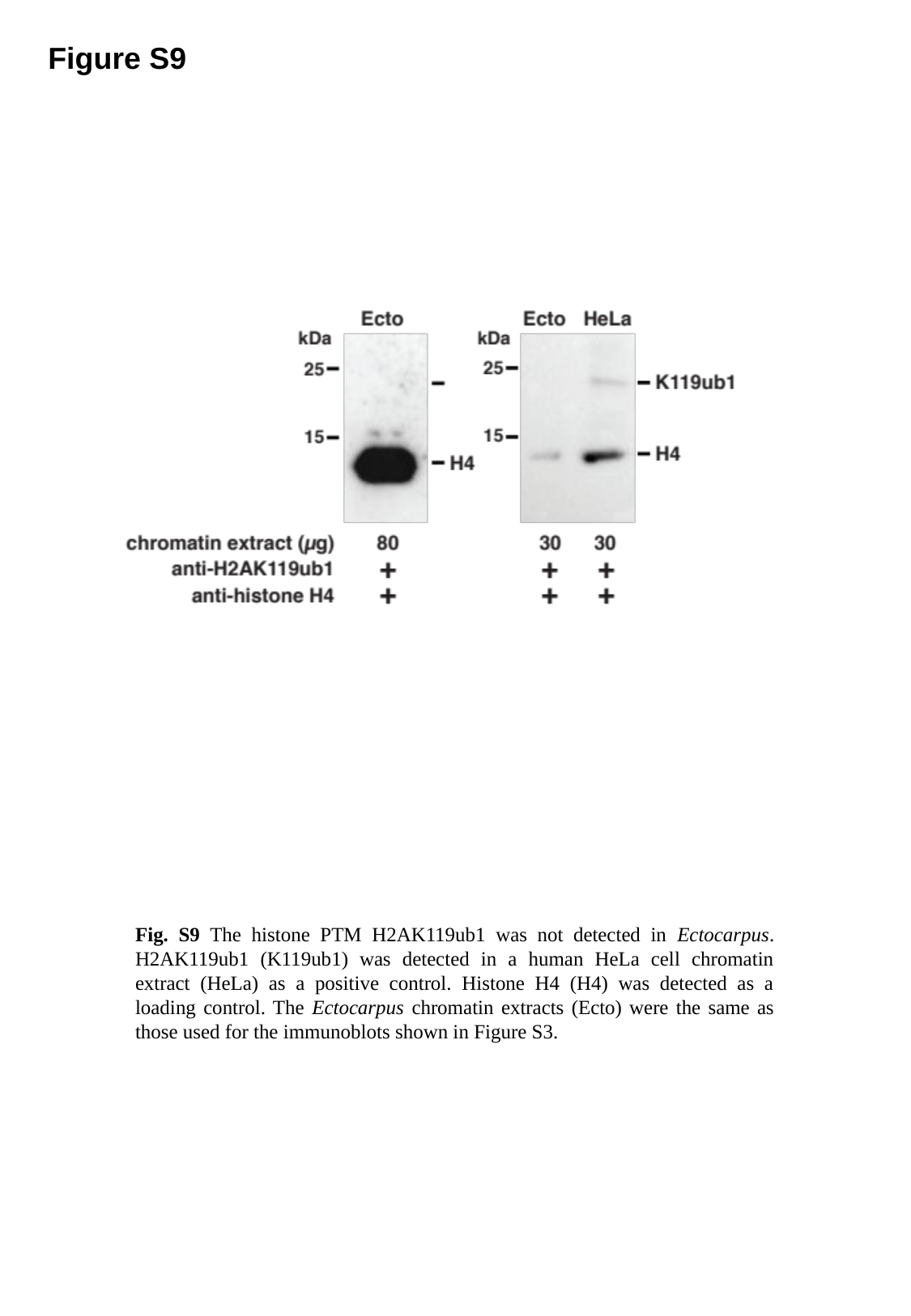

Figure S9
Fig. S9 The histone PTM H2AK119ub1 was not detected in Ectocarpus. H2AK119ub1 (K119ub1) was detected in a human HeLa cell chromatin extract (HeLa) as a positive control. Histone H4 (H4) was detected as a loading control. The Ectocarpus chromatin extracts (Ecto) were the same as those used for the immunoblots shown in Figure S3.

## Slide 8
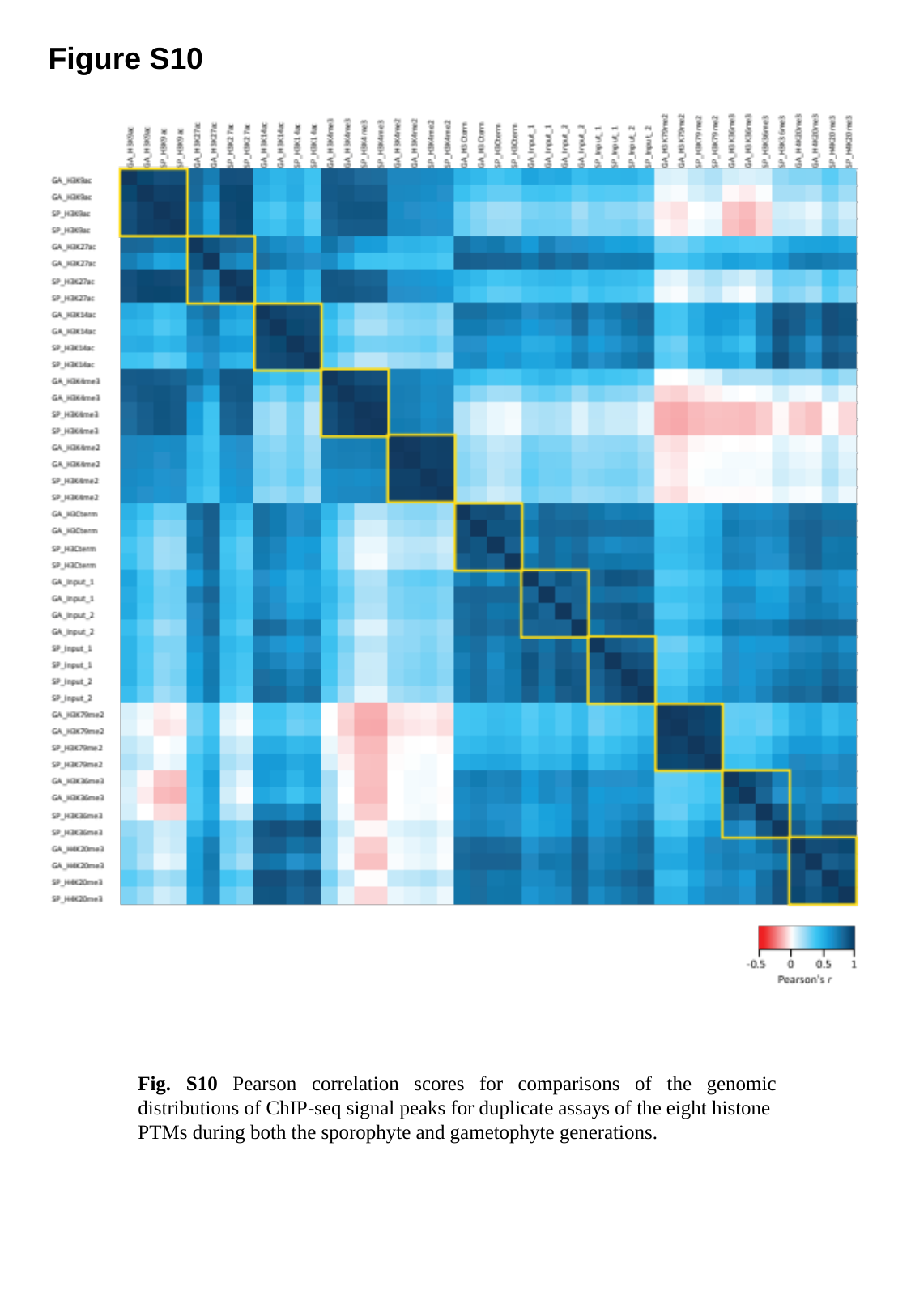

Figure S10
Fig. S10 Pearson correlation scores for comparisons of the genomic distributions of ChIP-seq signal peaks for duplicate assays of the eight histone
PTMs during both the sporophyte and gametophyte generations.
