# Supplementary material for: Histone modifications during the life cycle of the brown alga Ectocarpus
Source: Genome Biol. 2021 Jan 4;22:12. doi: 10.1186/s13059-020-02216-8 (PMC7784034; doi:10.1186/s13059-020-02216-8)
Supplement: Supplementary file 6 — Additional file 6: Table S2. Putative PTM writers and erasers in Ectocarpus sp. [file 13059_2020_2216_MOESM6_ESM.pdf]

Table S2. Putative PTM writers and erasers in *Ectocarpus* sp. Human and *Phaeodactylum tricornutum* (diatom) orthologues are also reported. Substrate specificity and biological functions are indicated based on what is known for humans and should be only considered as putative for *Ectocarpus* enzymes. NO CAT, no known category; ψ, probable pseudogene; v, inserted viral gene.

Table S2.1. Histone Lysine Acetyltransferases (KAT)

KAT	*Homo sapiens* [1]	*Ectocarpus* sp.	*Phaeodactylum tricornutum*	Substrate specificity [1]
GNAT Family				
KAT1	HAT1	Ec-01_007540	Phatr3_J54343	H4K5 and K12
KAT2A/B	GCN5	Ec-12_003920	Phatr3_J2957	H3K9, K14 and K18
?	ATAC2	No orthologues	No orthologues	H3K9, K14 and K18
KAT9	ELP3	Ec-12_002990	Phatr3_J50848	H3 and maybe H4
NO CAT		Ec-01_001170, Ec-01_005970, Ec-01_005990, Ec-01_006000, Ec-01_008410, Ec-02_003420, Ec-02_006170, Ec-05_004370, Ec-07_002520, Ec-08_002320, Ec-08_005180, Ec-08_005960, Ec-15_004510, Ec-16_003580, Ec-17_000040, Ec-17_000920, Ec-17_001110, Ec-22_002740, Ec-24_004370, Ec-28_000410		
MYST Family				
KAT5	TIP60	No orthologues	No orthologues	H4K5, K8, K12 and K16
KAT6A	MOZ	No orthologues	No orthologues	H3K9 and K14
KAT6B	MORF	No orthologues	No orthologues	H3K9 and K14
KAT7	HBO1	No orthologues	No orthologues	H4K5, K8 and K12
KAT8 stram_A	MOF	Ec-14_006220	Phatr3_J51406	H3K14 and K23, H4K16
KAT8 stram_B	MOF	Ec-22_002080	Phatr3_J3062	
p300/CBP Family				
KAT3A	CBP			Not discriminating
KAT3B	p300			Not discriminating
KAT3 stram_A		Ec-18_002450, Ec-04_001240	Phatr3_J45703, Phatr3_J54505, Phatr3_J45764	Not discriminating
KAT3 stram_B		Ec-04_005740	Phatr3_Jdraft292	Not discriminating

Table S2.2. Histone Lysine Methyltransferases (KMT)

KMT	Domains	*Ectocarpus* sp.	*Phaeodactylum tricornutum*	*Homo sapiens*	Substrate specificity
SET domain-containing KMTs					
MLL Family					
KMT2A-D	PWWP + ZF-PHD + BRD + FY-rich + SET + Post-SET	Ec-18_000480	Phatr3_J15937	MLL1, MLL2, MLL3, MLL4 [2]	H3K4 [2]
SUV39 *Family*					
KMT1A-B	CHROMO + AWS + SET + Post-SET	Ec-17_000690	No orthologues	SUV39H1, SUV39H2 [3]	H3K9 [3]
KMT1E-F	ZF-PHD + MBD + BRD + SET	Ec-12_006400	No orthologues	SETDB1, SETDB2 [3]	H3K9 [3]
SMYD Family					
SMYD	SET + MYND	Ec-04_001520	Phatr3_J1647, Phatr3_J43708	SMYD1-5 [3]	H3K4, H4K20 ? [3]
No human-related families					
NO CAT	AWS + SET	Ec-27_005100	No orthologues	No orthologues	Unknown
NO CAT	AWS + SET + Post-SET + ZF-PHD	Ec-05_003500	Phatr3_J6093	No orthologues	Unknown
NO CAT	SET + Post-SET	Ec-12_004700, Ec-14_005625, Ec-15_002050, Ec-15_002680, Ec-28_001240	Phatr3_J21456	No orthologues	Unknown
NO CAT	ZF-PHD + SET	Ec-12_006750ψ, Ec-12_006810, Ec-19_005100	No orthologues	No orthologues	Unknown
NO CAT	ZF-PHD + Pre-SET + SET	Ec-14_005310	No orthologues	No orthologues	Unknown
NO CAT	BRD + SET	Ec-12_006600	No orthologues	No orthologues	Unknown
NO CAT	SET + BRK + ZF-CW + CHROMO + TPR	Ec-12_000580	Phatr3_J44935	No orthologues	Unknown
KMTs with putative new functions					
Kinase-containing SET	AWS + SET + Post-SET + Cyclin-like + Kinase	Ec-06_008170	Phatr3_EG02211	No orthologues	Unknown
ADD-containing SET	ZF-ADD + Pre-SET + SET + Post-SET	Ec-22_000420	No orthologues	No orthologues	Unknown
Other SET					
NO CAT	SET	Ec-00_001310, Ec-01_004930, Ec-05_000850, Ec-06_002960, Ec-06_005230v, Ec-06_005320v, Ec-06_006470v, Ec-07_003020, Ec-07_004730, Ec-07_007100, Ec-08_006000, Ec-14_004620, Ec-14_004640, Ec-18_001770, Ec-19_004190, Ec-20_002460, Ec-26_000740, Ec-28_002300, Ec-14_005625	Phatr3_J39209, Phatr3_EG01652, Phatr3_J38974, Phatr3_J50541, Phatr3_J43311, Phatr3_J43708, Phatr3_EG01005, Phatr3_J43177, Phatr3_J48703, Phatr3_J24019		Unknown

NO CAT	SET + Rubisco LSMT	Ec-01_001070, Ec-06_004080, Ec-11_003630, Ec-12_007560, Ec-14_001980, Ec-14_003300, Ec-20_001330	Phatr3_J43946, Phatr3_J48815, Phatr3_J37749		Unknown
DOT1 domain-containing KMTs					
KMT4	DOT1	Ec-06_007110, Ec-12_004580, Ec-24_003550	Phatr3_J47512, Phatr3_J44757	DOT1 [4]	H3K79 [4]
KMT4_ecto	DOT1 + ZF-PHD	Ec-25_003090	No orthologues	No orthologues	H3K79 [4]

Table S2.3. Histone Arginine Methyltransferases (PRMT)

**RMT**	***Ectocarpus* sp.**	***Phaeodactylum tricornutum***	***Homo sapiens***
PRMT Family			
NO CAT	Ec-06_002620, Ec-14_006300, Ec-27_000650, Ec-27_005280	Phatr3_J17184, Phatr3_J54710, Phatr3_J44159, Phatr3_J45331, Phatr3_EG02379	PRMT1-9
PRMT5	Ec-10_005680	Phatr3_J16141	

Table S2.4. Histone Deacetylases (HDAC)

**HDAC**	***Ectocarpus* sp.**	***Phaeodactylum tricornutum***	***Homo sapiens* [5]**
Class I			
	Ec-15_004560, Ec-21_001720, Ec-28_001400	Phatr3_J49800, Phatr3_J43919, Phatr3_J51026	HDAC1-3, 8
Class II			
	Ec-05_003890, Ec-05_006370, Ec-06_004320, Ec-11_001830, Ec-10_001980	Phatr3_J4590, Phatr3_EG01943, Phatr3_J35869, Phatr3_J50482, Phatr3_J8891, Phatr3_J45431, Phatr3_J4423	HDAC4,5,7,9 HDAC6, 10
Class IV			
	Ec-14_000150, Ec-24_003590	Phatr3_J9278, Phatr3_J4821	HDAC11
Class III / Sirtuin Family			
	Ec-05_003910, Ec-17_002180, Ec-19_000750, Ec-21_005430, Ec-26_003780,	Phatr3_J8827, Phatr3_J16859, Phatr3_J12305, Phatr3_J45850, Phatr3_J52135, Phatr3_J21543, Phatr3_J39523	SIRT1-7

Table S2.4. Histone Lysine Demethylases (KDM)

KDM	Domains	*Ectocarpus* sp.	*Phaeodactylum tricornutum*	*Homo sapiens* [6]	Substrate specificity [6]
Lysine Specific Demethylases Family					
KDM1	Amine oxidase	Ec-10_001210, Ec-21_006430, Ec-24_001850	Phatr3_J51708, Phatr3_EG01090, Phatr3_J44106, Phatr_J48603	LSD1, LSD2	H3K4me1/2, H3K9me1/2
Jumonji-C Domain-containing Family					
KDM2	F-box + JmjC	Ec-21_004490	Phatr3_J42595	FBXL10, FBXL11	H3K36me1/2
KDM4D	JmjN + JmjC	Ec-22_001750	No orthologues	JMJD2	H3K9me1/2/3
KDM5A-B	JmjN +JmjC + ZF-C5HC2 + ARID + ZF-PHD	Ec-01_004230, Ec-21_005410	Phatr3_J48747	JARID1A-C	H3K4me2/3
KDM6A	TPR + JmjC	No orthologues	No orthologues	UTX	H3K27me2/3
KDM6B	JmjC	Ec-02_001410, Ec-02_001750, Ec-03_002440, Ec-07_006870, Ec-10_002180, Ec-10_005720, Ec-14_002420, Ec-14_004840, Ec-14_004970, Ec-15_003970, Ec-17_001710, Ec-21_001600, Ec-27_002280	Phatr3_J43557, Phatr3_J48473, Phatr3_J35781, Phatr3_J42595, Phatr3_EG01348	JMJD3	

References

[1] **Yang XJ. 2016.** Histone Acetyltransferases, Key Writers of the Epigenetic Language. In *Chromatin Signaling and Diseases*, Academic Press, Elsevier.

[2] **Gu B, Lee MG. 2013.** Histone H3 lysine 4 methyltransferases and demethylases in self-renewal and differentiation of stem cells. *Cell & Bioscience* **3**: 39.

[3] **Mozzetta C, Boyarchuk E, Pontis J, Ait-Si-Ali S. 2015.** Sound of silence: the properties and functions of repressive Lys methyltransferases. *Nature Reviews Molecular Cell Biology* **16**: 499-513.

[4] **Feng Q, Wang H, Ng HH, Erdjument-Bromage H, Tempst P, Struhl K, Zhang Y. 2002.** Methylation of H3-Lysine 79 Is Mediated by a New Family of HMTases without a SET Domain. *Current Biology* **12**: 1052-1058.

[5] **Lamberti MJ, Vera RE, Rumie Vittar NB, Schneider G. 2016.** Histone Deacetylases, the Erasers of the Code. In *Chromatin Signaling and Diseases*, Academic Press, Elsevier.

[6] **García MA, Fueyo R, Martínez-Ballás MA. 2016.** Lysine Demethylases: Structure, Function, and Disfunction. In *Chromatin Signaling and Diseases*, Academic Press, Elsevier.
