# Supplementary material for: Histone modifications during the life cycle of the brown alga Ectocarpus
Source: Genome Biol. 2021 Jan 4;22:12. doi: 10.1186/s13059-020-02216-8 (PMC7784034; doi:10.1186/s13059-020-02216-8)
Supplement: Supplementary file 7 — Additional file 7: Table S3. Presence or absence of histone post-translational modifications in seven species from diverse eukaryotic supergroups. [file 13059_2020_2216_MOESM7_ESM.pdf]

Table S3. Presence or absence of histone post-translational modifications in seven species from diverse eukaryotic supergroups. Probable equivalent PTMs at slightly different positions are also indicated. Not detected, not detected by either mass spectrometry or immunoblot; *, PTM only detected by immunoblot; †, the mark H3K27me3 was also weakly detected by immunoblot but further analysis indicated that it is not present in *Ectocarpus* (see text for details); No equivalent, the species in question has no amino acid residue equivalent to the *Ectocarpus* residue; Ac, acetylation; Me1, monomethylation; Me2, dimethylation; Me3, trimethylation; Pr, propylation; Ub, ubiquitination; -, not determined; Nt, N-alpha terminal.

**Histone residue**	***Ectocarpus* sp.**	***Phaeodactylum tricornutum* [1]**	***Thalassiosira pseudonana* [2]**	***Tetrahymena thermophila* [3,4]**	***Arabidopsis thaliana* [5–7]**	***Homo sapiens* [8–12]**	***Saccharomyces cerevisiae* [4,10,11,13]**
Histone H2A canonical							
S1	Ac(Nt)	_	_	_	_	Ac(Nt)	Ac(Nt)
K3	Ac	Ac	Not detected	No K3 equivalent	Not detected	No K3 equivalent	K4 Ac
K5	Ac	Ac, K7Ac	Not detected	Ac, K8 Ac, K10 Ac, K12 Ac	Ac	Ac, K9Ac	K7 Ac
Histone H2A.Z							
S1	Ac(Nt)	_	_	_	_	Not detected	_
K3	Ac	Ac	_	_	_	K4 Ac	_
K6	Ac	Ac	_	_	_	K7 Ac	_
K9	Ac	Ac	_	_	_	No K9 equivalent	_
K12	Ac	Ac	_	_	_	K11 Ac	_
K15	Ac	Ac	_	_	_	Not detected	_
K20	Ac	Not detected	_	_	_	K13 Ac	_
R38	Me1	Not detected	_	_	_	Not detected (R31)	_
Histone H2B							
K2	Ac	Ac	Ac	K3 not detected, Me3	No K2 equivalent	No K2 equivalent	K3 Ac
K6	Ac	Ac	Ac	K4 Ac	Ac	K5 Ac	Ac
K7	Ac	K6 Ac	K6 Ac	K5 not detected	K6 Ac	K5 Ac	Ac
K10	Ac	Ac	K11 Ac	No K10 equivalent	K11 Ac	K11 Ac, K12 Ac	K11 Ac
K13	Ac	Ac	K14 Ac	K12 not detected	K12 not detected	K15 Ac	K16 Ac
K14	Ac	Ac	K15 Ac	K13 not detected	K12 not detected	K16 Ac	Not detected
K34	Not detected	Ac	No K34 equivalent	No K34 equivalent	K32 Ac, K27 Ac	Not detected	K21 Ac, K22 Ac
K37	Not detected	K38 not detected	K38 Ac	K38 Not detected	K32 Ac	No K37 equivalent	Not detected

K47	Not detected	K48 Ac	K52 Ac, Me2	No K47 equivalent	K50 not detected	K47 Me1	K46 not detected
K107	Not detected	Ac	K103 Ac	No K107 equivalent	K105 not detected	K108 Me1	K108 Ac
K111	Ub	Ub	Ub not detected, Ac	Ub not detected, Ac	K143 Ub	K120 Ub	K123 Ub
Histone H3							
K4	Me2*, Me3*	Me1, Me2, Me3	Not detected	Not detected	Me1, Me2, Me3	Me1, Me3	Me1, Me2, Me3, Ac
K9	Ac	Ac	Ac	Ac	Ac	Ac	Ac
K9	Me1*, Me2*, Me3*	Me2*, Me3*	Not detected	Not detected	Me1, Me2*, Me3*	Me1, Me2, Me3	Me2, Me3
K14	Ac	Ac	Ac	Ac	Ac	Ac, Me1	Ac, Me2
K18	Ac	Ac	Ac	Ac	Ac	Ac, Me1	Ac, Me1
K23	Ac	Ac	Ac	Ac, Me1	Ac	Ac, Me1	Ac, Me1
K27	Ac	Ac	Ac	Ac	Ac	Ac	Ac
K27	Me2[†]	Me1, Me2, Me3	Me2	Me1, Me2, Me3	Me1, Me2, Me3	Me1, Me2, Me3	Me1, Me2, Me3
K36	Not detected	Ac	Ac	Ac	Ac	Not detected	Ac
K36	Me1, Me2, Me3	Me1, Me2, Me3	Me1, Me2, Me3	Me1	Me1, Me2, Me3	Me1, Me2, Me3	Me1, Me2, Me3
K56	Not detected	Ac	Not detected	Ac	Not detected	Ac, Me1, Me3	Ac
K79	Not detected	Ac	Ac	Not detected	Not detected	Ac	Not detected
K79	Me1, Me2	Me1, Me2	Me1, Me2, Me3	Me1	Not detected	Me1, Me2, Me3	Me1, Me2, Me3
K122	Not detected	Ac	Ac	Not detected	Not detected	Ac, Me1	Not detected
Histone H4							
S1	Ac(Nt)	_	_	_	_	Ac(Nt)	Ac(Nt)
K5	Ac	Ac	Ac	K4 Ac	Ac	Ac, Me1	Ac
K5	Pr	_	_	_	_	Pr	_
K8	Ac	Ac	Ac	K7 Ac	Ac	Ac	Ac, Me1
K12	Ac	Ac	Ac	K11 Ac	Ac	Ac	Ac, Me1
K16	Ac	Ac	Ac	K15 Ac	Ac	Ac	Ac
K20	Me1, Me2, Me3*	Ac, No methylation detected	Ac, No methylation detected	Me1	Ac, Me3	Me1, Me2, Me3	Me1, Me2
K31	Not detected	Ac	Not detected	Not detected	Not detected	Me2	Ac
R55	Not detected	Not detected	Not detected	Not detected	Not detected	Me1, Me2	Not detected
K59	Not detected	Ac	Ac	Not detected	Not detected	Not detected	Not detected
K59	Me1, Me2	Me1	Me1	Not detected	Not detected	Me1, Me2	Me1

K79	Me2, Me3	Me1, Me2, Me3	Me1, Me2, Me3	R77 Me1	Not detected	me2, K77 Me1	K77 Me1
K91	Not detected	Not detected	Ac	Not detected	Not detected	Ac	Not detected

References

1. Veluchamy A, Rastogi A, Lin X, Lombard B, Murik O, Thomas Y, et al. An integrative analysis of post-translational histone modifications in the marine diatom *Phaeodactylum tricornutum*. Genome Biol. 2015;16:102.

2. Rastogi A, Lin X, Lombard B, Loew D, Tirichine L. Probing the evolutionary history of epigenetic mechanisms: what can we learn from marine diatoms. AIMS Genet. 2.

3. Zhang C, Gao S, Molascon AJ, Wang Z, Gorovsky MA, Liu Y, et al. Bioinformatic and proteomic analysis of bulk histones reveals PTM crosstalk and chromatin features. J Proteome Res. 2014;13:3330–7.

4. Morris SA, Rao B, Garcia BA, Hake SB, Diaz RL, Shabanowitz J, et al. Identification of histone H3 lysine 36 acetylation as a highly conserved histone modification. J Biol Chem. 2007;282:7632–40.

5. Zhang K, Sridhar VV, Zhu J, Kapoor A, Zhu J-K. Distinctive core histone post-translational modification patterns in *Arabidopsis thaliana*. PLoS One. 2007;2:e1210.

6. Charron J-BF, He H, Elling AA, Deng XW. Dynamic landscapes of four histone modifications during deetiolation in *Arabidopsis*. Plant Cell. 2009;21:3732–48.

7. Mahrez W, Arellano MST, Moreno-Romero J, Nakamura M, Shu H, Nanni P, et al. H3K36ac Is an Evolutionary Conserved Plant Histone Modification That Marks Active Genes. Plant Physiol. 2016;170:1566–77.

8. Beck HC, Nielsen EC, Matthiesen R, Jensen LH, Sehested M, Finn P, et al. Quantitative Proteomic Analysis of Post-translational Modifications of Human Histones. Mol Cell Proteomics. American Society for Biochemistry and Molecular Biology; 2006;5:1314–25.

9. Tan M, Luo H, Lee S, Jin F, Yang JS, Montellier E, et al. Identification of 67 histone marks and histone lysine crotonylation as a new type of histone modification. Cell. 2011;146:1016–28.

10. Zhao Y, Garcia BA. Comprehensive Catalog of Currently Documented Histone Modifications. Cold Spring Harb Perspect Biol. 2015;7:a025064.

11. Hole K, Van Damme P, Dalva M, Aksnes H, Glomnes N, Varhaug JE, et al. The human N-alpha-acetyltransferase 40 (hNaa40p/hNatD) is conserved from yeast and N-terminally acetylates histones H2A and H4. PloS One. 2011;6:e24713.

12. Huang H, Sabari BR, Garcia BA, Allis CD, Zhao Y. SnapShot: Histone Modifications. Cell. 2014;159:458-458.e1.

13. Valero ML, Sendra R, Pamblanco M. Tandem affinity purification of histones, coupled to mass spectrometry, identifies associated proteins and new sites of post-translational modification in Saccharomyces cerevisiae. J Proteomics. 2016;136:183–92.
